# Proton Conductivity in Photomagnetic Cu^II^_2_-[M^IV^(CN)_8_] Frameworks (M = Mo^IV^ and W^IV^) Facilitated by the Tetracarboxy-Derivative of Cyclam

**DOI:** 10.1021/acs.inorgchem.4c05576

**Published:** 2025-04-07

**Authors:** Mateusz Reczyński, Maciej Pazera, Michał Magott

**Affiliations:** Faculty of Chemistry, Jagiellonian University in Kraków, Gronostajowa 2, 30-387 Kraków, Poland

## Abstract

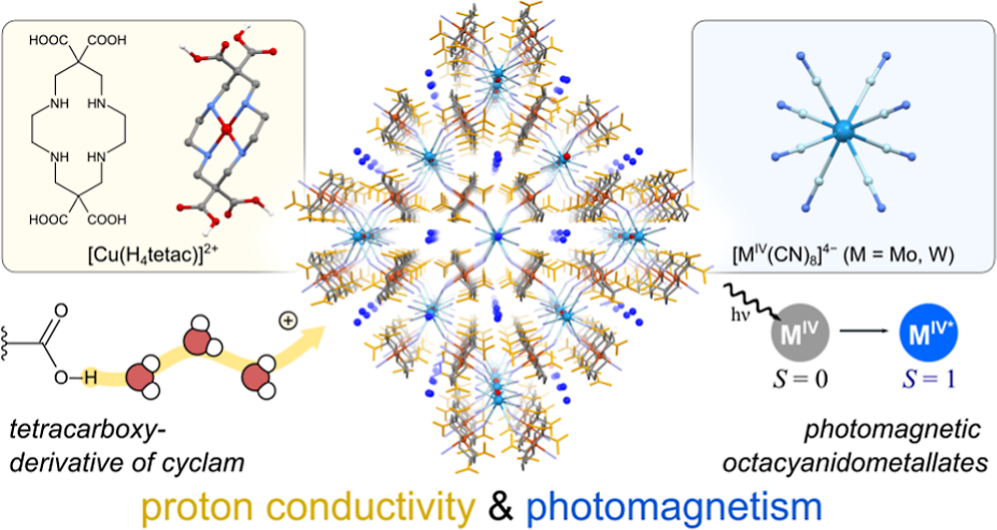

Two 3D bimetallic cyanido-bridged coordination polymers, [Cu^II^(H_4_tetac)]_2_[M^IV^(CN)_8_]·4H_2_O (M = Mo^IV^ (**1**) and W^IV^ (**2**)), based on copper(II) complex of tetracarboxylic-derivative of cyclam, H_4_tetac (=1,4,8,11-tetrazacyclotetradecane-6,6,13,13-tetracarboxylic acid), have been synthesized and characterized in terms of structure, proton conductivity, and the photomagnetic effect. The isostructural compounds crystallize in the polar *Fdd*2 space group and present a diamond-like topology. The carboxylic groups in the structure yield proton conductivities of 5.4(3) × 10^–7^ S cm^–1^ (**1**) and 8.6(3) × 10^–7^ S cm^–1^ (**2**) at 298 K and 98% relative humidity. A strong humidity dependence of conductivity and activation energy values of 0.34 eV (**1**) and 0.36 eV (**2**) indicate the Grotthuss mechanism of proton transport that is mediated by crystallization water molecules. In the ground state, **1** and **2** are paramagnets due to Cu^II^ (*S* = 1/2) centers separated by diamagnetic [M^IV^(CN)_8_]^4–^ anions (*S* = 0). Upon 450 nm light irradiation at 10 K, both compounds show a photomagnetic response. The Mo^IV^ system shows higher photoconversion rates, while the W^IV^ analogue exhibits full reversibility. Compounds **1** and **2** are the first examples of cyanido-bridged assemblies combining proton conductivity and the photomagnetic effect, advancing the reticular chemistry of cyanido-bridged frameworks.

## Abstract 2

Two 3D bimetallic cyanido-bridged coordination polymers, [Cu^II^(H_4_tetac)]_2_[M^IV^(CN)_8_]·4H_2_O (M^IV^ = Mo^IV^ (**1**) and W^IV^ (**2**)), were synthesized and characterized for their structural, conductive, and photomagnetic properties. These compounds exhibit proton conductivities of 5.4 × 10^−7^ (**1**) and 8.6 × 10^−7^ S cm^−1^ (**2**) at 298 K and 95% RH. Both compounds are paramagnetic, and upon 450 nm light irradiation, both exhibit a photomagnetic response related to spin transitions on M^IV^ centers from *S* = 0 to *S* = 1.

## Introduction

A constant increase in energy consumption constitutes one of the most important issues facing our civilization. Energy production based on fossil fuels entails the problem of pollution and degradation of the natural environment. Turning to greener energy sources is necessary, and it may be achieved by the development of nonemissive technologies, such as proton membrane fuel cells or high-capacity batteries to store energy from renewable but intermittent sources.^1,2^ Both approaches require highly efficient solid-state electrolytes and proton conductors. Currently, proton-exchange membranes and electrolytes are based on ceramics, oxides, or organic polymers such as Nafion.^3,4^ An interesting alternative to those materials is coordination polymers (CPs) and metal–organic frameworks (MOFs). Proton-conducting MOFs and CPs may be obtained as materials with well-defined crystal structures via rational design, and they achieve conductivity (σ) as high as 10^–1^ to 1 S cm^–1^.^5−7^ Lim and Kitagawa have recently reviewed design strategies leading to proton-conducting MOFs, which include impregnation structures with protic cations (H_3_O^+^ and NH_4_^+^), the functionalization of organic linkers (−COOH, –SO_3_H, and –PO_4_H_2_) or metal centers (aqua ligands and imidazole), the inclusion of protic guest molecules (H_2_O, imidazole, and H_2_SO_4_), and the introduction of defects in the structure.^6^

Similar design approaches may be applied to other CP classes, such as cyanido-bridged CPs. These compounds are very efficient platforms for creating all-in-one materials combining several functionalities, including magnetism, optical phenomena, porosity, and electrical properties.^8,9^ The most recognizable ones are Prussian blue analogues (PBAs), which have been intensively studied as potential electrode materials for developing high-performance batteries.^10,11^ However, compared to that of MOFs, proton conductivity in CN-bridged CPs is rarely investigated. The few examples reported so far show great potential for proton conducting multifunctional systems. The main source of proton conductivity in these materials is crystallization water molecules present in their structure.^12,13^ Likewise, the presence of aqua ligands coordinated to open sites of metal nodes in defected PBAs resulted in room temperature (RT) superionic conductivity (∼10^–3^ S cm^–1^) combined either with RT ferromagnetic ordering^14^ or second-harmonic generation.^15^ Moreover, impregnation of CN-bridged assemblies with protic cations (H_3_O^+^, NH_4_^+^, and 4-aminopirydynium cation) was proven to be a sufficient way to achieve H^+^ conductivity.^12,16^ In our previous studies, we used that approach in anionic CPs based on [Ni(cyclam)]^2+/3+^ complexes (cyclam = 1,4,8,11-tetraazacyclotetradecane) and polycyanidometallates [M(CN)_*n*_]^*m*−^. We have obtained bimetallic coordination chains, H_3_O[Ni^III^(cyclam)][M^II^(CN)_6_]·5H_2_O (M = Ru^II^ and Os^II^), which exhibit dehydration-triggered electron-transfer between Ni^III^ and M^II^ centers and superionic conductivity of 1.1 × 10^–3^ S cm^–1^ at 295 K and 100% of relative humidity (RH).^13^ In this case, the presence of H_3_O^+^ cations leads to high σ values, and most probably, the change in their distribution upon dehydration drives the electron-transfer process. In another study, we have introduced NH_4_^+^ cations into two-dimensional (NH_4_)_2_[Ni^II^(cyclam)]_3_[Nb^IV^(CN)_8_]_2_·21H_2_O coordination layers through electrolyte-assisted synthesis,^17^ which proved to be useful for the introduction of other cations into the Ni_3_Nb_2_ framework.^18^ The layered structure of this material is crossed with channels filled with crystallization water and NH_4_^+^ cations, which creates suitable conduction pathways for proton transport and results in σ of ∼4 × 10^–5^ S cm^–1^ (295 K, 100% RH). Additionally, the microporous and flexible structure of the material leads to significant humidity-dependence of its magnetic characteristics related to changes in the interlayer distances and Ni–N≡C–Nb bridge geometry.

All of the above, though successful, show that the design of proton conductivity in CN-bridged systems is limited to the incorporation of water molecules or protic cations. Therefore, we decided to investigate another approach, which is the use of ligands functionalized with acidic groups (−COOH and –SO_3_H) as a source of mobile protons. For this purpose, we used a carboxy-derivative of cyclam: 1,4,8,11-tetrazacyclotetradecane-6,6,13,13-tetracarboxylic acid (=H_4_tetac), whose complexes should act, similarly to the original macrocycle, as linear building blocks forming microporous networks with [M(CN)_*n*_]^*m*−^ as well as be a source of protons. The H_4_tetac ligand is synthesized on a copper template as the [Cu^II^(H_4_tetac)]^2+^ complex,^19,20^ which we have directly combined with [M^IV^(CN)_8_]^4–^ (M = Mo^IV^ and W^IV^) aiming at microporous H^+^-conductive materials. In addition, by using the [M^IV^(CN)_8_]^4–^ blocks, we intended to incorporate a photomagnetic effect into a proton-conducting system. The possibility of manipulation of materials’ magnetic behavior with light in photomagnetic systems is particularly interesting due to a wide range of possible applications arising from it, such as light-induced switching, data storage and processing, or sensing. There are several strategies for introducing the photomagnetic response to molecular systems based on metal complexes. These include the utilization of spin-crossover active complexes of iron(II) exhibiting the light-induced excited spin-state trapping (LIESST) phenomenon^21−23^ or cyanido-bridged bimetallic redox couples exhibiting charge-transfer-induced spin-transition upon light irradiation.^24−27^ The [M^IV^(CN)_8_]^4–^ anions enable both (i) the photoinduced charge transfer, which often occurs for Cu^II^–Mo^IV^ systems with the formation of the Cu^I^–Mo^V^ state,^28−33^ and the LIESST phenomenon at M^IV^ centers (*S* = 0), which leads to the induction of the M^IV*^ (*S* = 1) state.^34−38^ Recently, it has also been shown that the photomagnetic response may be achieved by photodissociation of CN^–^ ligand in the K_4_[Mo^IV^(CN)_8_]·2H_2_O salt.^37^ In this work, we report the synthesis and characterization of two novel three-dimensional CPs, [Cu^II^(H_4_tetac)]_2_[M^IV^(CN)_8_]·4H_2_O (M = Mo (**1**), W (**2**)), which combine proton conductivity, facilitated by the H_4_tetac ligand, and the photomagnetic effect introduced with the [M^IV^(CN)_8_]^4–^ anions.

## Experimental Section

### Materials

All of the reagents and solvents were purchased from available vendors (Merck, Idalia) and used as supplied. The precursors [Cu(H_4_tetac)](ClO_4_)_2_·4H_2_O,^19,20^ K_4_[Mo(CN)_8_]·2H_2_O,^39^ and K_4_[W(CN)_8_]·2H_2_O^40^ were synthesized following the literature procedures.

#### [Cu(H_4_tetac)]_2_[Mo(CN)_8_]·4H_2_O (**1**)

A solution of K_4_[Mo(CN)_8_]·2H_2_O (6.2 mg, 0.0125 mmol) in 0.25 M HCl (0.5 mL) was added to a solution of [Cu(H_4_tetac)](ClO_4_)_2_·4H_2_O (17.8 mg, 0.025 mmol) in 0.25 M HCl (4.5 mL). First pink needle-shaped crystals of **1** formed after 10 min. The pink solution was left overnight for crystallization. The crystals were vacuum-filtered, washed with distilled water, and air-dried. Yield: 75%. EA. Calculated for C_36_H_56_Cu_2_N_16_O_20_Mo: C, 34.43; H, 4.49; N, 17.84. Found: C, 34.26; H, 4.60; N, 18.23. IR (cm^–1^). 1229 s, 1272 m, 1453 m, 1728 vs, 2117 vs, 2465 br, 2554 br, 2886 m, 3248 s, 3555 m, 3640 m.

#### [Cu(H_4_tetac)]_2_[W(CN)_8_]·4H_2_O (**2**)

The tungsten analogue was synthesized analogously to **1** using K_4_[W(CN)_8_]·2H_2_O (7.3 mg, 0.0125 mmol) instead of K_4_[Mo(CN)_8_]·2H_2_O. The pink needle-shaped crystals of **2** were collected after 1 day by vacuum filtration, washed with water, and air-dried. Yield: 78%. EA. Calculated for C_36_H_56_Cu_2_N_16_O_20_W: C, 32.17; H, 4.20; N, 16.68. Found: C, 31.89; H, 4.31; N, 17.03. IR (cm^–1^). 1228 s, 1272 m, 1453 w, 1728 vs, 2108 vs, 2463 br, 2555 br, 2886 w, 3248 s, 3557 w, 3642 w.

### Structure Determination

Single-crystal diffraction data were collected at 100 K on a Bruker D8 Venture diffractometer equipped with a Mo Kα radiation source (λ = 0.71073 Å) and a Photon III detector. To avoid dehydration, the crystals of **1** and **2** were taken directly from the mother liquor and placed in a protective layer of NVH immersion oil (Cargille). Unit cell determination, measurement strategy calculation, and data collection were performed with the Bruker APEX4 software. The data examination performed with XPREP suggested noncentrosymmetric space group *Fdd*2. The structures were solved with the intrinsic phasing method using SHELXT,^41^ and the models were refined against *F*^2^ with SHELXL software^42^ in *Fddd* and *Fdd*2 space groups. The latter solution was refined as an inversion twin, which gave less disordered models with better *R* indices than the centrosymmetric one. In the case of **2**, the unit cell of the initial model was transformed to match the model of **1**. All non-hydrogen atoms were refined as anisotropic. The C–H and N–H hydrogen atoms were placed in idealized geometry and refined with *U*_iso_(H) = 1.2 *U*_eq_ for the C or N atoms using the riding model. The O–H hydrogen atoms were located from the Fourier differential map, taking into account suitable H-bonds and the length of C–O bonds in the carboxylic groups. In most cases, the unconstrained refinement gave reasonable bounding geometries. In the final refinement, the H geometry was constrained to ideal values bond angles (DFIX and DANG) and refined using the riding model (AFIX 4) with *U*_iso_(H) = 1.2 *U*_eq_(O). Deposition nos. 2379259 (**1**), 2379258 (**2**), and 2379257 ([Cu(H_4_tetac)](ClO_4_)_2_·4H_2_O) contain the supplementary crystallographic data for this paper. These data are provided free of charge by the joint Cambridge Crystallographic Data Centre and Fachinformationszentrum Karlsruhe Access Structures service www.ccdc.cam.ac.uk/structures.

### Physical Measurements

Elemental analysis was performed on an Elementar Vario Cube CHNS analyzer. The infrared spectra were collected on a Thermo Scientific Nicolet iN 10 FTIR microscopic spectrometer for samples pressed to a BaF_2_ window. The UV–vis–NIR electronic spectra were collected on a Shimadzu UV-3600i Plus spectrophotometer in diffuse transmittance mode for sample suspension in paraffin oil pressed between two quartz windows. The thermogravimetric analysis was performed in the flow of dry N_2_ on a Netzsch TG209 F1 Libra analyzer in the range of 21–300 °C with a heating rate of 1 K min^–1^. The water sorption isotherms were collected at 295 K on an SMS DVS Resolution apparatus in the range of 0–96% of RH with a 2% step and a d*m*/d*t* = 0.002% min^–1^ mass stability threshold. Differential scanning calorimetry profiles were collected with a Netzsch DSC214 Polyma calorimeter in the 173–323 K range with heating and cooling rates of 10 K min^–1^. Powder diffraction patterns for samples packed into glass capillaries were collected on a Bruker D8 Advance ECO diffractometer equipped with Cu Kα (λ = 1.5406 Å) radiation source. The Le Bail analysis concerning the peak profiles and unit cell parameters for RT powder diffraction patterns was performed with EXPO2014 software.^43^

### Impedance Studies

The ionic conductivity studies were performed with a BioLogic MTZ-35 electrochemical impedance analyzer equipped with an ITS temperature chamber and a controlled environment sample holder. The data were collected using a four-probe method for pelletized samples [5 mm in diameter and thickness of 0.18 mm (**1**) and 0.34 mm (**2**)] placed between two gold electrodes. The data were recorded in the frequency range of 10 MHz to 10 Hz. The RH in the sample cell was stabilized using an L&C HG-100 RH generator. First, the RH relation of ionic conductivity was investigated at 298 K in the 30–98% RH range. At each RH step, measurement cycles were performed every 20 min until there was no significant change in the signal for at least three subsequent cycles. Second, the temperature dependence of conductivity was investigated at 80% RH in the temperature range of 290–303 K. The impedance data were fitted with suitable equivalent circuits using the BioLogic ECLab software.

### Magnetic and Photomagnetic Studies

The magnetic susceptibility studies were performed on a Quantum Design MPMS3 Evercool magnetometer in the temperature range 2–300 K and for the magnetic field up to 7 T. Dry samples for bulk magnetic susceptibility measurements were packed into foil bags and sealed to avoid dehydration in the magnetometer chamber. The diamagnetic contributions were subtracted from the data. Photomagnetic measurements were performed on samples placed in a thin layer between two layers of Scotch tape and mounted in a plastic straw. The sample was inserted into the magnetometer at 250 K, and then, the sample chamber was pumped to avoid dehydration. The photoirradiation was performed with a 450 nm laser diode with a light power of 10 mW cm^–2^ (measured at the sample). The sample mass and diamagnetic corrections were determined by comparison to bulk measurements.

## Results and Discussion

### Synthesis and Structure

**Figure 1:**
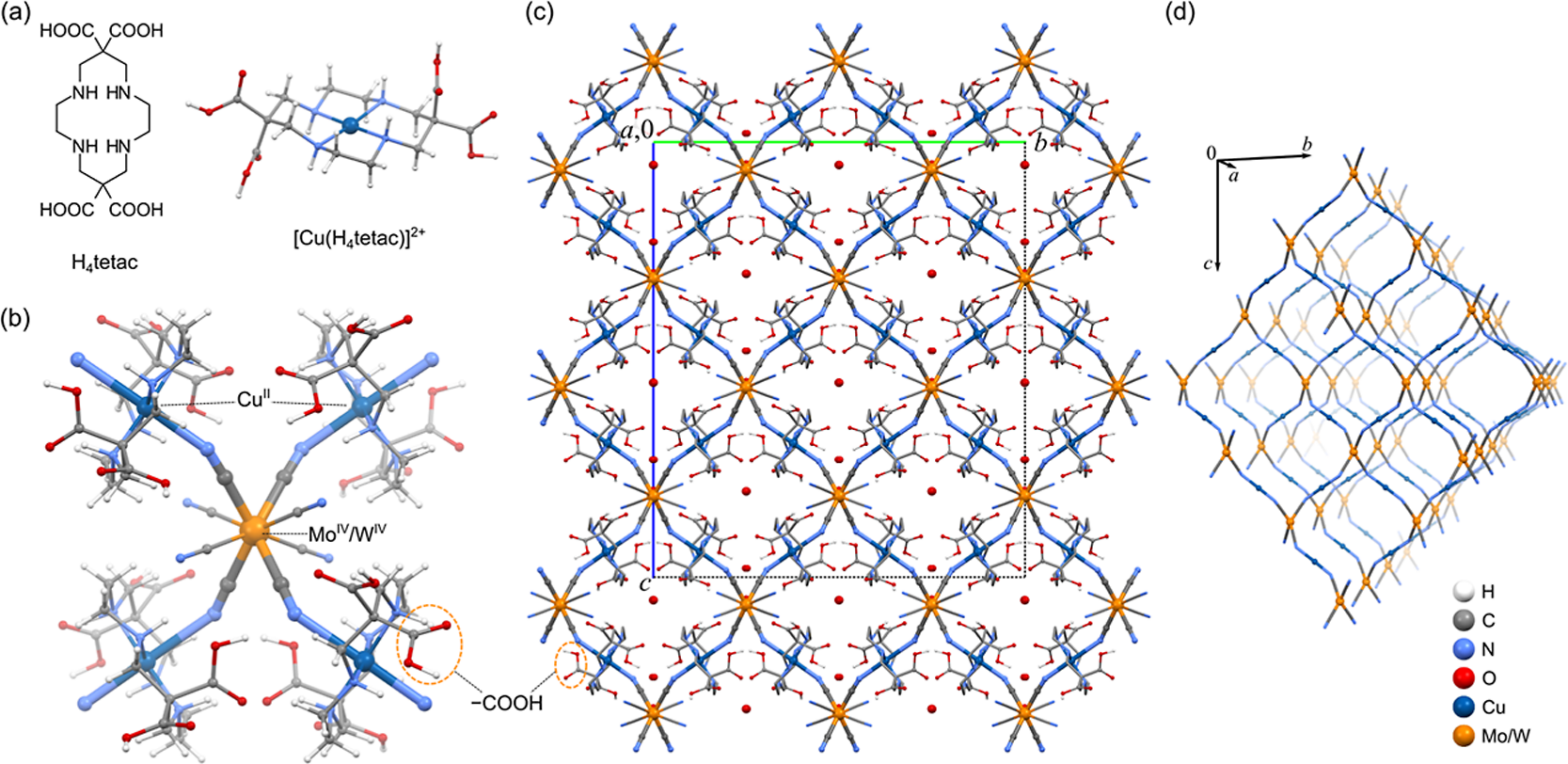
Structures of **1** and **2** at 100 K: (a) the structures of the H_4_tetac ligand and the [Cu(H_4_tetac)]^2+^ building block, a fragment of the structure with the octacyanidometallate(IV) node connecting four [Cu(H_4_tetac)]^2+^ moieties (b), a packing diagram viewed along axis *a* (c), and a schematic representation of the diamond-like topology of the CPs (d). Some hydrogen atoms were omitted for clarity.

The starting complex, [Cu^II^(H_4_tetac)](ClO_4_)_2_·4H_2_O (H_4_tetac = 1,4,8,11-tetrazacyclotetradecane-6,6,13,13-tetracarboxylic acid), was synthesized and fully characterized before use according to the method published by Xin et al.^19,20^ with minor modifications, which led to the same product (see Figures S1–S3). Although the complex was synthesized over 30 years ago, its crystal structure has not been described yet. The complex crystallizes in the *P*1̅ space group, and its asymmetric unit consists of half of the [Cu(H_4_tetac)](ClO_4_)_2_ complex and two crystallization water molecules. The H_4_tetac ligand, a 6,6,13,13-tetracarboxylic-derivative of cyclam (Figure 1a), *N*-coordinates the equatorial positions of Cu^2+^ center, while the axial ones are occupied by ClO_4_^–^ anions. The Cu^2+^ center presents a distorted octahedron geometry with significant elongation of axial bonds (continuous shape measure CShM = 1.234).^44^ All of the carboxylic groups are protonated, which is reflected in the lengths of the C=O (1.21 and 1.22 Å) and C–O (∼1.31 Å) bonds (Figure S1). However, the O–H bonds are longer than 1.15 Å with H atoms shifted toward O atoms of crystallization water molecules, which form strong H-bonds with the carboxylic groups. From the structural point of view, the [Cu(H_4_tetac)]^2+^ complex (Figure 1a) is a promising building block for the construction of proton-conducting CPs since it has two axial sites suitable for the bridge formation and the macrocyclic ligand is functionalized with four proton donor groups.

Direct combination of [Cu^II^(H_4_tetac)](ClO_4_)_2_ with [Mo^IV^(CN)_8_]^4–^ or [W^IV^(CN)_8_]^4–^ in acidic solution leads to the formation of pink needle-shaped crystals of the [Cu^II^(H_4_tetac)]_2_[Mo^IV^(CN)_8_]·4H_2_O (**1**) or [Cu^II^(H_4_tetac)]_2_[W^IV^(CN)_8_]·4H_2_O (**2**), respectively. The acidic conditions were used to ensure the protonation of the carboxylic groups of the ligand. Yet the acid concentration should not be excessive because it may cause decarboxylation of the geminal acid, which leads to complexes of 6,13-dicarboxy-cyclam derivative.^20^ The products were characterized by elemental analysis and FTIR spectroscopy, while their phase stability and purity were conformed with differential scanning calorimetry and powder XRD (Figures S4–S7 and Table S1).

Compounds **1** and **2** are isostructural and crystallize in orthorhombic space group *Fdd*2. Since the structural differences between **1** and **2** are minor, the structures are described collectively with detailed structural parameters listed in Tables S2–S3. The compounds are three-dimensional cyanido-bridged CPs of diamond-like topology (Figure 1). The asymmetric units consist of half of an [M^IV^(CN)_8_]^4–^ anion with the metal center placed on a 2-fold axis, one [Cu(H_4_tetac)]^2+^ complex in general position, one water molecule in general position, and two-halves of H_2_O placed on 2-fold axes (Figure S8). The [M^IV^(CN)_8_]^4–^ complexes present a triangular dodecahedron geometry (*D*_2*d*_; CShM = 0.299 (Mo), 0.279 (W)). They act as nodes in the framework and *N*-coordinate the axial sites of the [Cu(H_4_tetac)]^2+^ complexes through four of their CN^–^ ligands (Figure 1b). While the equatorial Cu–N bonds to the macrocycle are uniform (2.01–2.03 Å), the axial bonds differ strongly in length (2.41–2.45 Å for Cu1–N1 and 2.70–2.74 Å for Cu1–N2; Table S3). This results in a distorted octahedral geometry of the Cu centers (CShM = 1.694 (**1**), 1.637 (**2**)). Moreover, the M–CN–Cu bridges are significantly bent, which is best described by the C–N–Cu angles of ca. 149 and 141° for N1 and N2 atoms, respectively. This bonding arrangement leads to a three-dimensional diamond-like coordination framework (Figure 1c,d), similar to [Ni(cyclam)]-based systems.^45^ The observed topology differs from previous reports for cyclam-based system [Cu^II^(cyclam)][Mo^IV^(CN)_8_]·10H_2_O,^46,47^ forming a two-dimensional square grid motif, which suggests that functionalization of the macrocycle with –COOH groups led to a higher dimensionality of the network. Similarly to [Cu^II^(H_4_tetac)](ClO_4_)_2_, all of the COOH groups are protonated, which is reflected in the C–O(H) (1.27–1.33 Å) and C=O (1.17–1.22 Å) bond lengths (Table S3). Interestingly, the orientation of the COOH groups on both sides of the macrocycle is inequivalent, which lowers the symmetry of the complex and may be the reason for the polar point group of the systems.

**Figure 2:**
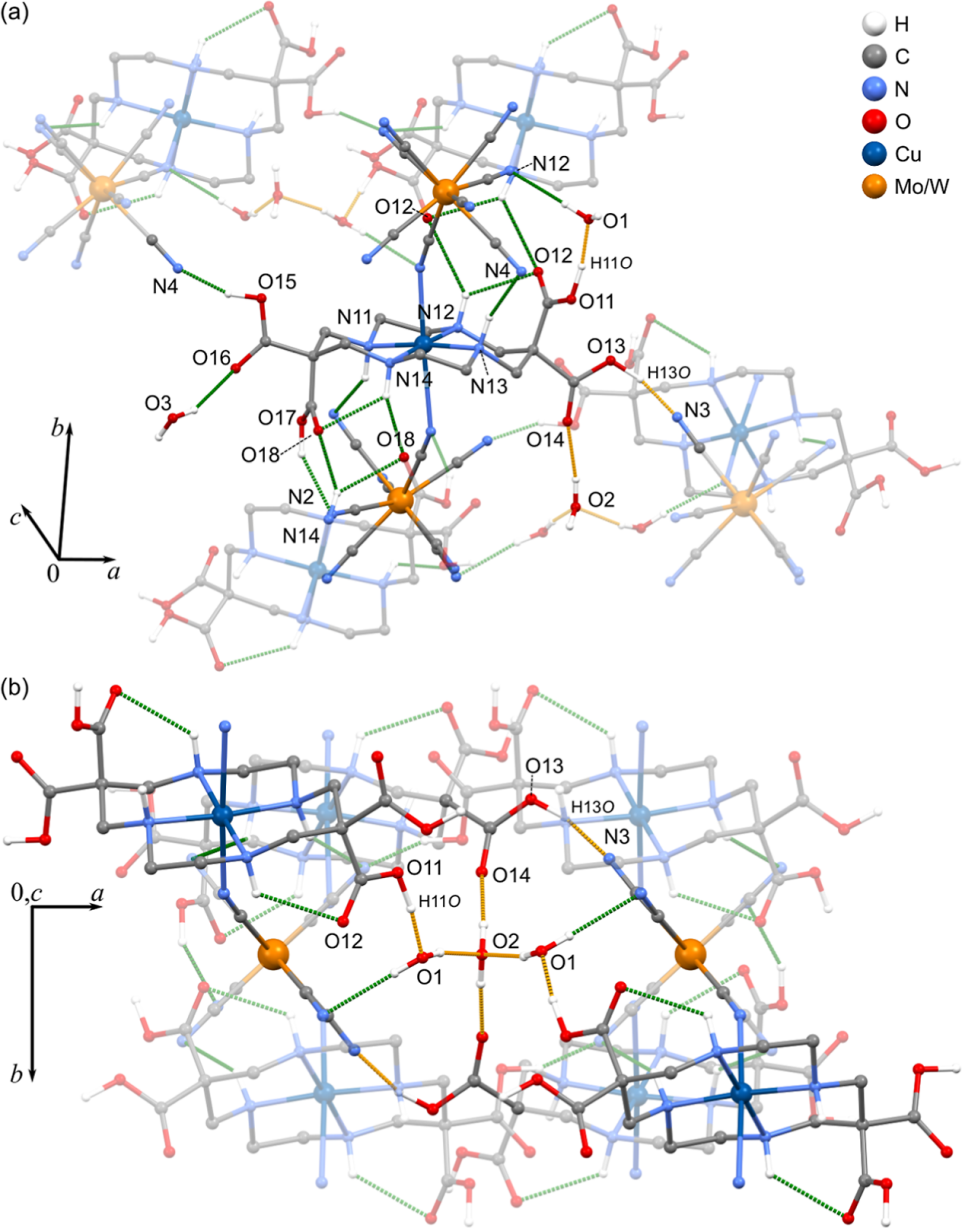
Selected hydrogen bonds in the structures of **1** and **2** formed by the H_4_tetac ligand (a) and crystallization water molecules (b). The orange contacts are potential paths of proton hopping according to the Grotthuss mechanism. Hydrogen atoms not involved in significant H-bonds were omitted for clarity.

The network presents discrete cavities partly filled with crystallization water molecules (Figure S9). The solvent-accessible volume constitutes around 6% of the unit cell volume. The pores are narrow, with the limiting and largest pore diameters of 0.2 and 0.3 nm, respectively.^48,49^ Based on the single-crystal analysis, crystallization water molecules occupy only the cavities surrounded by carboxylic groups and terminal CN^–^ ligands with which they form hydrogen bonds (Figure 2 and Table S4). Voids confined by the ligand’s ethylene fragments (−CH_2_–CH_2_−) are empty or occupied by highly disordered H_2_O molecules due to less effective H-bonding of aliphatic parts of the ligand. Hydrogen bonds are formed within the coordination framework by N–H and COOH groups and CN^–^ ligands, which stabilize the structure (Figure 2). Three of the ligand’s carboxylic groups are donors of O–H···N bonds to CN^–^ ligands and do not form an extensive network of H-bonds.

The remaining group is a donor of an O–H···O bond with H_2_O molecules in the cavity, which form a short H-bond chain motif with a symmetry-related COOH group. Since this motif composes an alternate connection of proton donors and acceptors (Figure 2b, orange contacts), it constitutes a potential pathway for water-mediated proton hopping according to the Grotthuss mechanism.^50,51^

### Proton Conductivity

The proton conductivity (σ) in **1** and **2** was investigated with electrochemical impedance spectroscopy for the pelletized powder samples. The resistance (*R*) of the materials was obtained by fitting the equivalent circuits to the impedance data, which in the most general case includes the contributions of grain interior (*R*_gr_/*Q*_gr_; *Q*—constant phase element), grain boundary (*R*_br_/*Q*_br_), and the electrode polarization process (*Q*_el_). The total resistance of the material was calculated as the sum of *R*_gr_ and *R*_br_, and the conductivity values were calculated as σ = *d*/*A*·*R*, where *d* is the pellet thickness, *A* is the contact surface, and *R* is the total resistance.

**Figure 3:**
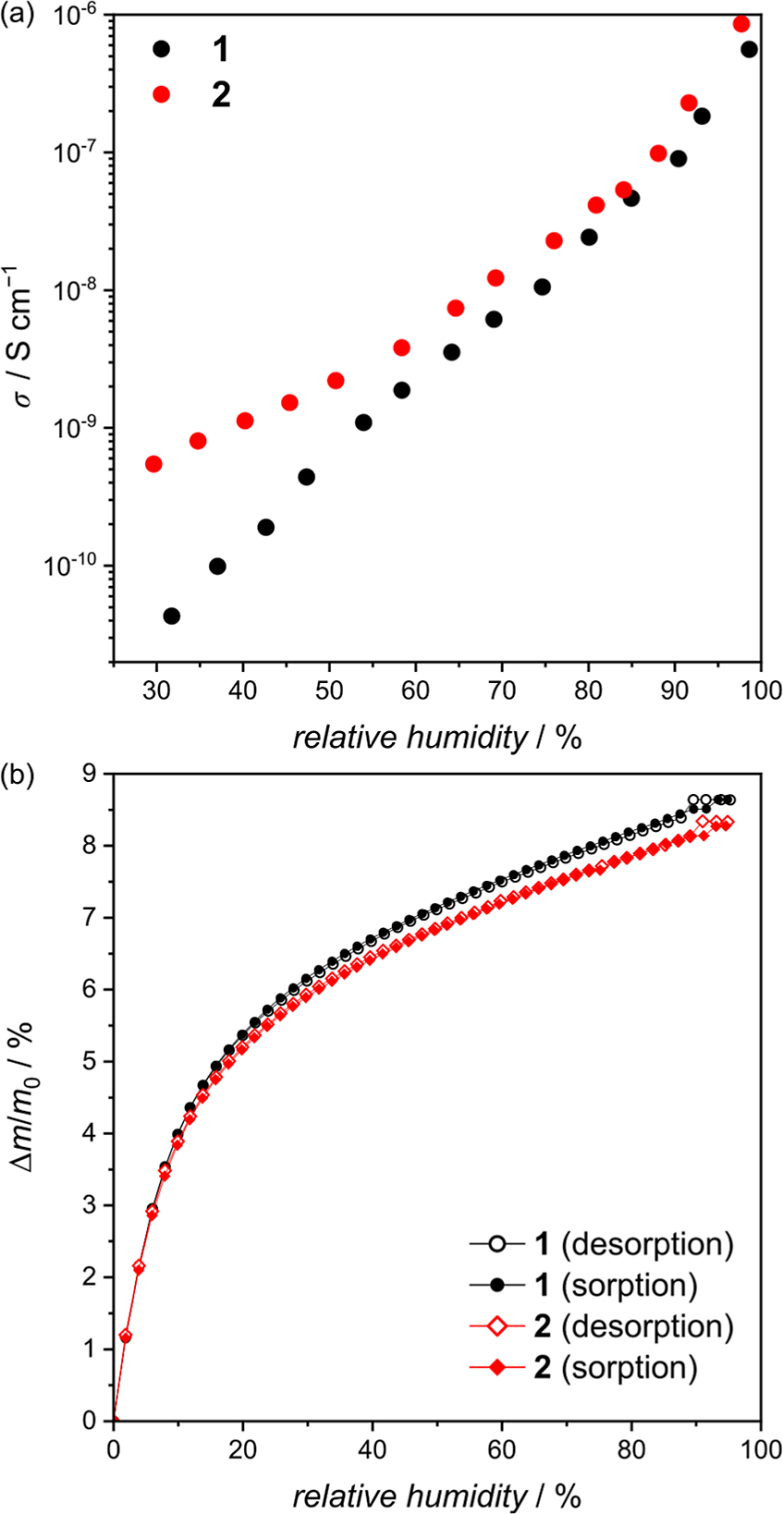
RH dependence of conductivity (a) and water vapor sorption isotherms (b) for **1** (black) and **2** (red) at 298 K.

Initially, the dependence of conductivity on RH was investigated at 298 K (Figures 3a and S10 and S11). At high RH of ca. 98%, σ reaches values of 5.4(3) × 10^–7^ S cm^–1^ and 8.6(3) × 10^–7^ S cm^–1^ for **1** and **2**, respectively. Upon humidity reduction, the conductivity drastically decreases, and around 30% RH σ drops to 4.3(3) × 10^–11^ and 5.5(2) × 10^–10^ S cm^–1^ for **1** and **2**, respectively. The strong relationship between conductivity and humidity proves that the crystallization-water molecules mediate proton transport in these materials. To investigate this relation further, we measured water vapor adsorption isotherms for **1** and **2** at 298 K (Figure 3b). Both compounds present type I adsorption profiles characteristic of microporous solids.^52^ Sorption and desorption processes are reversible, indicating that the coordination network is rigid and structural changes related to water sorption are minor. Initially, in the range 0–20% RH, the water uptake increases fast but not abrupt. At higher RH, sorption slows down, and above 40% RH, the water uptake grows linearly without a plateau. Such characteristics suggest that the voids where H-bonds are formed (higher affinity to water) are filled first (0–30% RH), while at higher RH sorption occurs mostly on the surface. Moreover, the pore-filling process may be limited by diffusion of water molecules through rather narrow cavities. Around 25–30% RH, the water content is 4 mol mol^–1^ (water uptake of ca. 0.060 g g^–1^), which agrees with the single crystal models and elemental analysis results. This also corresponds well with the fact that below 30% RH, the values of resistance of the samples are very high, and a reliable determination of σ was not possible. This proves that filling the micropores is essential for constituting the conduction pathways. Further water sorption at higher humidity fills the gaps in the conduction pathways, leading to a strong increase in σ. Finally, at 96% RH, the water uptake reaches ∼0.085 g g^–1^, which most probably comes from the sorption on the surface of the material. Interstitial water may both stabilize the conduction paths within the grains and constitute new ones between them.

**Figure 4:**
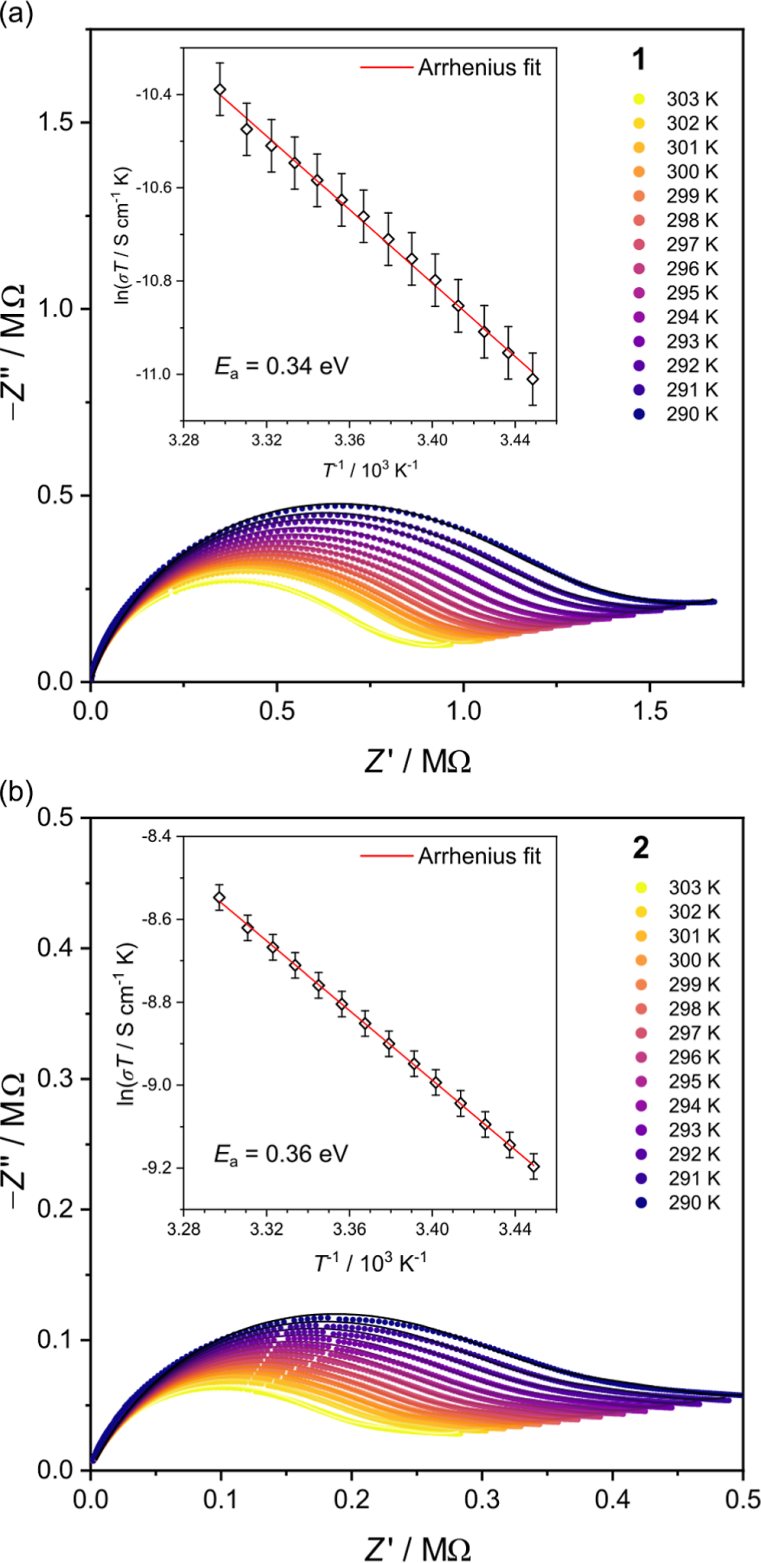
Temperature dependence of conductivity in **1** (a) and **2** (b): the Nyquist plots recorded at 290–303 K at 80% RH and ln(σ*T*) vs *T*^–1^ plots (inserts) with the fits of the Arrhenius law (red lines). The solid lines on Nyquist plots depict the best fitting results obtained for the (*R*_gr_/*Q*_gr_) + (*R*_br_/*Q*_br_) + *Q*_el_ equivalent circuit.

The temperature-dependent impedance studies (Figure 4) were conducted on a sample conditioned at 80% RH at 298 K. Data were collected at around RT (290–303 K) to avoid the dehydration of the materials, as observed on the TGA profiles in dry N_2_ (Figure S12). As expected, the temperature dependence shows an increase in σ with increasing temperature. The Arrhenius relation, σ*T* = σ_0_ exp(−*E*_a_/*kT*) (*k*—the Boltzmann constant), was fitted to the ln(σ*T*) vs *T*^–1^ plots and the activation energy values of *E*_a_ = 0.34 and 0.36 eV were determined for **1** and **2**, respectively. The *E*_a_ values imply that the conductivity in both compounds occurs with the Grotthuss mechanism, in which the protons migrate by hopping on paths set by the H-bond network.^6,50^ The stability of the compounds in dynamic water vapor sorption and conductivity studies was proven by PXRD measurements that indicated no significant changes in the structure (Figure S13).

Although the compounds contain 8 protic groups per formula unit (the concentration of –COOH is 0.011 mol cm^–3^), the observed conductivity values are rather low (less than 1 × 10^–6^ S cm^–1^) compared to other COOH-based molecular proton conductors in similar conditions (Table S5).^53−58^ This originates from the materials’ structure in which most of the carboxyl groups form direct H-bonds to the coordination network, which limits the mobility of the protons. In the case of the COOH groups forming H-bonds with H_2_O molecules, the H-bond network is limited to a finite chain motif of three H_2_O molecules confined within narrow pores (Figure 2b). In this environment, the protons may hop between –COOH groups and H_2_O molecules or associate with another COOH group through the carbonyl O(14) atom. The latter requires tautomerization of the carboxylic group with instant protonation of one of the terminal CN(3) ligands. Besides providing protons, the carboxylic groups sterically reduce the volume and dimensionality of pores as well, decreasing their availability for water molecules. Therefore, despite the high concentration of protic groups, the lack of a long-range H-bond network and strong interactions of protons with the framework results in overall low conductivity values.

### Magnetic and Photomagnetic Studies

Direct current magnetic properties of **1** and **2** were investigated in the function of temperature (2–300 K) at 1 kOe and magnetic field (up to 70 kOe) at 2.0 K (Figures S14). The susceptibility and temperature product (χ*T*) value in both compounds is constant in the 300–5 K range and equals 0.79 cm^3^ K mol^–1^, which is expected for a paramagnetic state originating from weakly interacting Cu^II^ centers (*g* = 2.0 and *S* = 1/2) separated by diamagnetic [M^IV^(CN)_8_]^4–^ species. Below 5 K, the χ*T* slightly decreases, which suggests weak antiferromagnetic interactions between the Cu^II^ centers. The magnetization at saturation at 70 kOe reaches values of 1.94 and 1.92 Nβ for **1** and **2**, respectively.

**Figure 5:**
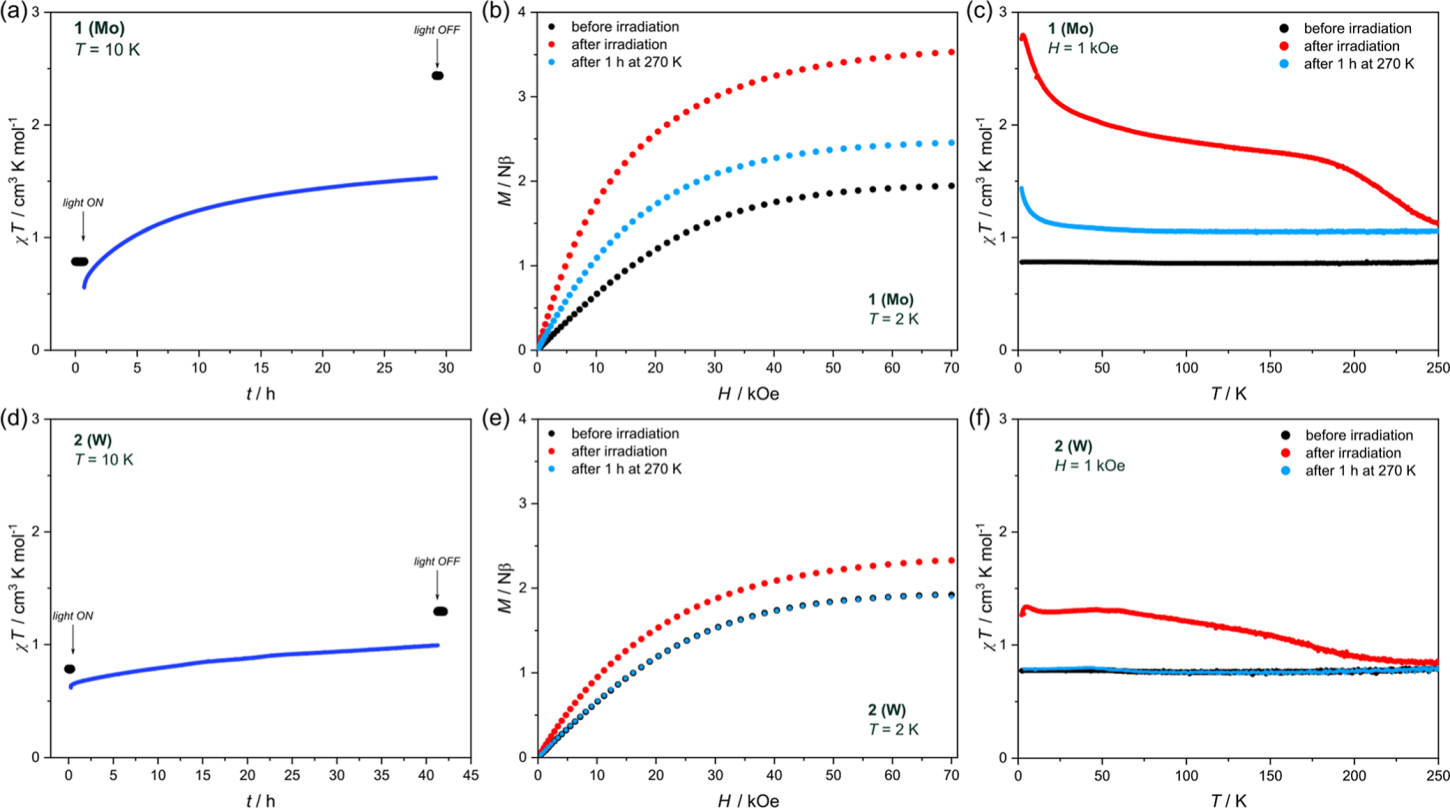
Photomagnetic properties of **1** and **2**: χ*T*(*t*) dependences recorded upon the 450 nm-light irradiation at 10 K and 1 kOe (a,d), field dependence of magnetization (b,e) at 2 K, and temperature dependence of χ*T* (c,f) at 1 kOe for samples before irradiation (black circles), after 450 nm light irradiation (red circles), and after 1 h thermalization at 270 K (light blue circles).

Prior to photomagnetic studies, the electronic spectra of compounds **1** and **2** were investigated (Figure S15). The spectra of the compounds are composed of (i) broad absorption bands (∼460–700 nm) originating from ligand-field absorption in the Cu^II^ complexes, (ii) ligand-field bands (460 ∼ 300 nm), and (iii) metal-to-ligand charge-transfer bands (<300 nm) in [M^IV^(CN)]^4–^ complexes.^35,37,59^ Therefore, photomagnetic properties of **1** and **2** were investigated by irradiation with 450 nm light, which falls in the range of ligand-field bands of the octacyanidometallates(IV). Upon light irradiation at 10 K, both **1** and **2** show significant evolution of magnetization (Figure 5a,d). The χ*T* increases from 0.79 to 2.44 cm^3^ K mol^–1^ in the case of **1** and from 0.79 to 1.29 cm^3^ K mol^–1^ for **2** (Figure 5b,e). The light irradiation has a significant impact on magnetization at saturation recorded at 2 K for both compounds, as well. In the case of **1**, magnetization at 70 kOe increases from 1.94 Nβ before irradiation to 3.53 Nβ after irradiation was finished (Figure 5b). In principle, this effect may result from two different processes: light-induced metal-to-metal charge transfer in the Cu^II^–Mo^IV^ pair, leading to the Cu^II^–Mo^V^–Cu^I^ system,^28−33^ or from the spin transition of the molybdenum(IV) to the *S* = 1 state.^34−38^ The light-induced increase in magnetization (ca. 1.6 Nβ per Cu_2_Mo formula unit) must result from the appearance of new unpaired electrons in the system, as even in the case of the ferromagnetically coupled *S* = 1 Cu^II^–Mo^V^ pair (*S*_Cu_ = 1/2, *S*_Mo_ = 1/2), the magnetization at a saturation value of 3.53 Nβ would correspond to an unlikely *g*_iso_ value larger than 3.5. Thus, we ascribe the photomagnetic effect in **1** to the *S* = 0 to *S* = 1 spin transition centered on Mo^IV^. In principle, the appearance of the additional *S* = 1 moiety in the system for *g*_Mo_ = 2.0 should correspond to approximately 2 Nβ increase in the magnetization, slightly more than the 1.6 Nβ observed experimentally. However, this difference can be explained by the less than 100% photoconversion efficiency as well as the zero-field splitting (ZFS) effect, which was previously observed for the photoinduced *S* = 1 state of [Mo^IV^(CN)_8_]^4–^.^37^

The expected χ*T* value for two noninteracting *g*_Cu_ = 2.0 and *S*_Cu_ = 1/2 centers and one *g*_Mo_ = 2.0 and *S*_Mo_ = 1 center would be *C* = *N*μ_B_^2^(2*g*_Cu_^2^*S*_Cu_(*S*_Cu_ + 1) + *g*_Mo_^2^*S*_Mo_(*S*_Mo_ + 1))/3*k*_B_ = 1.75 cm^3^ K mol^–1^. That is approximated well by the experimental χ*T* value, which after irradiation equals 1.76 cm^3^ K mol^–1^ at 150 K (Figure 5b, red curve), suggesting only weak superexchange interactions between Cu^II^ and high-spin Mo^IV^ centers, which are overcome by thermal energy at this temperature. Below 150 K, the experimental χ*T* value increases with a decrease in the temperature, reaching 2.79 cm^3^ K mol^–1^ at 3.3 K. The attempts to quantitively describe this behavior failed, as the full model should include the superexchange coupling *J*_Cu–Mo_, dipole–dipole interactions between Cu^II^ centers (which are separated in space by only 7.5–7.6 Å), and ZFS parameter (*D*) of high-spin Mo^IV^ centers, leading to the overparameterization of the fit. Nonetheless, the positive sign of *J*_Cu–Mo_ > 0 can be deduced from the previously mentioned increase of χ*T* associated with a decrease in the temperature. Despite the direct superexchange interaction pathways and cyanide connectivity in three dimensions, no sign of long-range magnetic ordering is observed down to 2 K, probably due to the long Cu^II^···NC-Mo^IV^ contacts, which limit the orbital overlap and strength of the resulting superexchange coupling.

Heating the photoirradiated **1** above 180 K results in a distinct decrease in the χ*T* value (Figure 5c), suggesting thermal relaxation of the high-spin Mo^IV^ centers. However, even after 1 h of sample thermalization at 270 K, the χ*T* does not return to the initial value (Figure 5c, red and blue curve, respectively). The reversibility of the transformation is not improved by heating the sample to 300 K (Figure S16, purple curve). This suggests that photoinduced changes triggered in **1** by blue light irradiation should be considered rather as an irreversible photo-anneal than the fully reversible photomagnetic switching.^60^

In the case of **2**, magnetization at 70 kOe increases from 1.92 Nβ before irradiation to 2.33 Nβ after irradiation (Figure 5e). This behavior most likely also results from the light-induced formation of *S* = 1 tungsten(IV) centers,^35,38^ although the observed low-spin to high-spin conversion efficiency is significantly smaller than in the case of **1**, and thus some degree of metal-to-metal charge transfer (MMCT) cannot be excluded. However, heating the sample to 270 K restores the initial magnetic characteristics (Figure 5e,f) rendering the photoinduced changes in **2** fully reversible. This is not unusual, as reversible photomagnetic switching of octacyanotungstate(IV) was previously observed in frameworks in which octacyanomolybdate(IV) analogues demonstrate only a partially reversible effect.^61−63^ Since [Mo^IV^(CN)_8_]^4–^ and [W^IV^(CN)_8_]^4–^ present almost identical structural features in **1** and **2** and have almost equal energy of electronic transitions in the UV–vis range,^59^ the origin of improved reversibility of photomagnetic transition in tungsten-based assemblies is most likely associated with the different transient structures of excited electronic states, which for now remains unclear. Nevertheless, **2** constitutes the second example of the photomagnetic copper(II)-octacyanotungstate(IV) system, with the observed photoinduced magnetization increase of 65% at 10 K vastly surpassing the 3% effect reported previously for the K_4_{[Cu^II^(ida)]_2_[W^IV^(CN)_8_]}·4H_2_O system (ida^2–^ = iminodiacetate).^64^

## Conclusions

In conclusion, for the first time, we successfully combined proton conductivity with a photomagnetic effect in two newly synthesized cyanido-bridged CPs, **1** and **2**. We achieved that by combining the copper(II) complex of the tetracarboxy derivative of cyclam, H_4_tetac, and octacyanidometallates(IV) (Mo^IV^ and W^IV^). We proved that using the COOH-functionalized macrocyclic ligand is a feasible design for proton conductors in CN-bridged systems. Although we achieved a high concentration of protic groups in the structure, the conductivity values are not proportionally large, which originates from the lack of an extensive hydrogen bonding network. Nevertheless, proton conductivity may be improved by the design of more open coordination networks with larger water-accessible space, either by changing the [M(H_4_tetac)]^2+^–polycyanidometallate stoichiometry or by using a less bulky dicarboxylic derivative of cyclam. Both approaches are currently being studied. The results reported here show great potential for research on the photomagnetic effect in the rarely studied systems composed of Cu^II^ and [W(CN)_8_]^4–^. Though the photoconversion in **2** (W^IV^) is lower than in **1** (Mo^IV^), it exhibits a higher conversion rate than previously reported for Cu^II^–W^IV^ and better reversibility than its Mo^IV^ analogue. Achieving better reversibility of the photomagnetic effect may lead to photoswitchable magnetic systems with higher durability, which we aim for in our current research.

## Supporting Information Available

Additional synthetic details, FTIR spectra, UV–vis spectra, powder diffraction data, additional crystallographic data, thermogravimetric data, differential scanning calorimetry, humidity-dependent Nyquist diagrams, and additional magnetic data of **1** and **2** (PDF)
