# Supplementary material for: Proton Conductivity in Photomagnetic CuII2-[MIV(CN)8] Frameworks (M = MoIV and WIV) Facilitated by the Tetracarboxy-Derivative of Cyclam
Source: Inorg Chem. 2025 Apr 7;64(15):7397–406. doi: 10.1021/acs.inorgchem.4c05576 (PMC12015808; doi:10.1021/acs.inorgchem.4c05576)
Supplement: Supplementary file 1 — ic4c05576_si_001.pdf [file ic4c05576_si_001.pdf]

Supporting information for

Proton Conductivity in Photomagnetic Cu^{II}_2-$[M^{IV}(CN)_8]$ Frameworks ($M = Mo^{IV}$, W^{IV}) Facilitated by the Tetracarboxy-Derivative of Cyclam

Mateusz Reczyński,* Maciej Pazera, and Michał Magott[a]

Faculty of Chemistry, Jagiellonian University in Kraków, Gronostajowa 2, 30-387 Kraków, Poland.

*E-mail: mateusz.reczynski@uj.edu.pl

Table of Contents

Synthesis and characterization of $[Cu(H_4tetac)(ClO_4)_2]\cdot 4H_2O$ (H_4tetac = 1,4,8,11-tetrazacyclotetradecane-6,6,13,13-tetracarboxylic acid)	S2-S4
Basic characterization of **1** and **2** (FTIR spectra, DSC profiles, powder diffraction patterns, and Le Bail analysis)	S4-S7
Additional crystallographic data for **1** and **2** (asymmetric units, structure parameters, selected bond lengths and angles, hydrogen-bond geometry, packing and void diagrams)	S7-S11
Nyquist diagrams recorded for **1** and **2** at different relative humidity conditions at 298 K	S12
Thermogravimetric profiles for **1** and **2**	S13
Comparison of PXRD patterns for **1** and **2** before and after impedance and sorption experiments	S14
Comparison of conductivities and activation energy in selected COOH-based proton conductors	S15
Magnetic properties of **1** and **2** in bulk	S15
Solid-state UV-vis spectra of **1**, **2**, and $[Cu(H_4tetac)](ClO_4)_2\cdot 4H_2O$	S16
Additional magnetic data for **1** recorded in photomagnetic experiment	S16
References	S17

Synthesis and characterization of [Cu(H_4tetac)(ClO_4)$_2$]·4H_2O (H_4tetac = 1,4,8,11-tetrazacyclotetradecane-6,6,13,13-tetracarboxylic acid)

[Cu(H_4tetac)(ClO_4)$_2$]·4H_2O was synthesized following the literature procedure via Mannich condensation on the Cu-complex template.[1] The procedure included the isolation of an intermediate product of the tetraethyl ester, [Cu(tetest)](ClO_4)$_2$ (**S1**; tetest = tetraethyl 1,4,8,11-tetrazacyclotetradecane-6,6,13,13-tetracarboxylate), followed by its hydrolysis in alkaline conditions.

[Cu(tetest)](ClO_4)$_2$ (**S1**). Ethylenediamine (7.5 g, 0.125 mol) was slowly added to an ethanolic solution of $CuCl_2$·2H_2O (8.5 g, 0.05 mol; 500 ml) and the mixture was heated under reflux for 30 min in a two-necked flask. Upon the addition of ethylenediamine, the mixture changed color from green to purple. A mixture of diethyl malonate (0.188 mol, 30 g) and formaldehyde (20 g, 37% w/w aqueous solution, 0.25 mol) diluted with ethanol (100 ml) was added to the mixture dropwise over 1 hour. The solution was refluxed for a further 40 hours, resulting in the darkening of its color. Next, the mixture was rotary evaporated and the oily residue was dissolved in warm water (50 ml), and treated with concentrated $HClO_4$ (70% w/w, 50 ml). The solution turned red and a pink precipitate of **S1** appeared. The solution was refrigerated overnight to complete precipitation. The pink product was filtered off, washed with a little cold water followed by ethanol, and air dried. The raw powder product was recrystallized from water/acetonitrile (5:3 v/v) to obtain a crystalline product. Yield: 5.3 %. EA. Calculated for $C_{22}H_{40}Cl_2CuN_4O_{16}$: C 35.18, N 7.46, H 5.37 %. Found: C 35.37, N 7.34, H 5.35 %. M = 751.0 g mol^{-1}. IR [cm^{-1}]. ν(C=O) 1736s, 1749sh; ν(N-H) 3252m, 3220w.

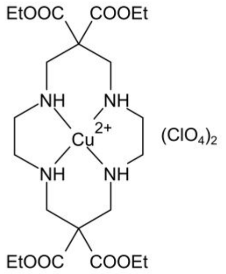

[Cu(tetest)](ClO_4)$_2$ (**S1**)

[Cu(H_4tetac)(ClO_4)$_2$]·4H_2O. S1 (2.25 mmol, 1.69 g) was dissolved in an aqueous 0.25 M NaOH solution (100 ml) and heated to near boiling for 3 hours. Acetonitrile was added to help it dissolve (up to 5 ml) if the solid kept floating on the surface. Upon heating the purple solution became brighter. The mixture was rotary evaporated to reduce its volume by 80 %, acidified with $HClO_4$ (70 % w/w, 30 ml), and refrigerated. The dark red crystalline product was filtered off, washed with a little cold water followed by ethanol, and air dried. Single crystals suitable for X-ray diffraction were selected from the reaction mixture. Yield: 1.41 g, 88.1 %. EA. Calculated for $C_{14}H_{32}Cl_2CuN_4O_{20}$: C 23.65, N 7.88, H 4.54 %. Found: C 23.45, N 7.97, H 4.68 %. M = 710.9 g mol^{-1}. IR [cm^{-1}]. ν(C=O) 1697s, 1723s; ν(N–H) 3226m, 3251m; ν(O–H) 3557s(br).

Structure determination for [Cu(H_4tetac)(ClO_4)$_2$]·4H_2O. The single-crystal diffraction data were collected at 100 K on a Bruker D8 Quest ECO diffractometer equipped with Mo Kα radiation source (λ = 0.71073 Å). The crystals were selected directly from the synthetic mixture and covered with a protective layer of NVH oil. Unit cell determination, measurement strategy, and data collection were performed with the Bruker APEX3 software. The structures were solved with the intrinsic phasing method using SHELXT and the models were refined against F^2 with SHELXL software. All non-hydrogen atoms were refined as anisotropic. The C-H and N-H hydrogen atoms were placed in the idealized geometry and refined with U_{iso}(H) = 1.2 U_{eq} of the of C or N atoms using the riding model. The O-H

hydrogen atoms were located from the Fourier differential map taking into account suitable H-bonds and the length of C-O bonds in the carboxylic groups. The geometry of water molecules was constrained to ideal values of bonds and angles (DFIX, DANG) with $U_{\mathrm{iso}}(\mathrm{H}) = 1.2\ U_{\mathrm{eq}}(\mathrm{O})$. The H atoms of the -COOH groups were refined without geometrical restraints. They are significantly shifted towards O atoms of H-bonded water molecules, which indicates that the H atoms are shared between the molecules. This resulted in alerts A concerning the elongated O-H bonds and short inter D-H...H-D distances. The geometry of corresponding H-bonds indicates strong H-bonds between -COOH and water molecules. The alerts are addressed in the CIF file with the validation response form. Crystal data: $C_{14}H_{32}Cl_2CuN_4O_{20}$, M_r = 710.87 g mol^{-1}, triclinic, space group P−1, a = 8.0084(3) Å, b = 8.9789(4) Å, c = 9.7943(4) Å, α = 83.067(2)°, β = 81.687(2)°, γ = 67.475(1)°, V = 642.07(5) Å^3, Z = 1, F(000) = 367.0, D_x = 1.838 g cm^{-3}. Refinement data: $R[F^2 > 2\sigma(F^2)]$ = 0.039, $wR(F^2)$ = 0.090, and S = 1.06 for 2326 independent reflections with $I > 2\sigma(I)$. CCDC deposition number: 2379257.

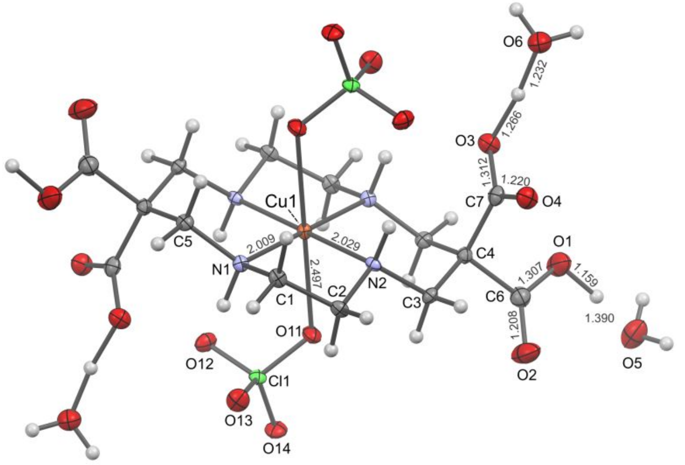

Figure S1. The structure of the $[Cu(H_4tetac)(ClO_4)_2]\cdot 4H_2O$. The atoms comprising an asymmetric unit are labelled and selected distances are given. The anisotropic displacement ellipsoids are drawn with 50% probability.

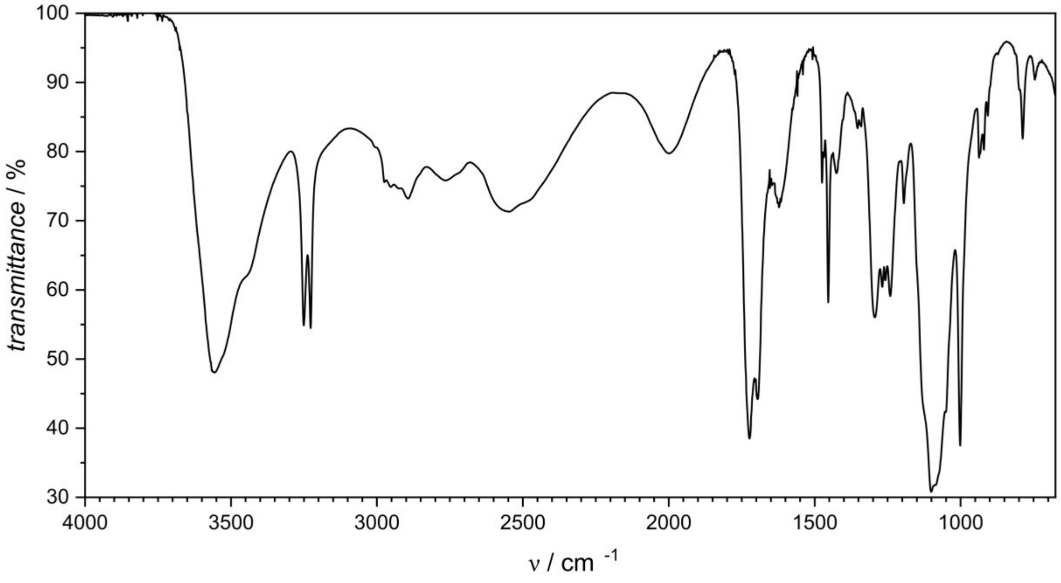

Figure S2. FT-IR spectrum of $[Cu(H_4tetac)(ClO_4)_2]\cdot 4H_2O$.

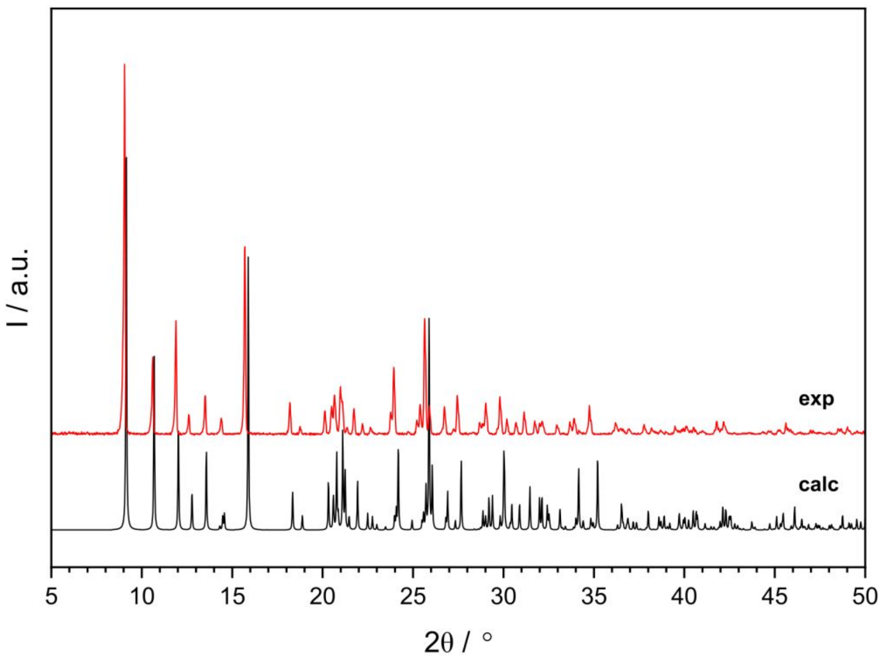

Figure S3. A comparison of a powder XRD pattern for $[Cu(H_4tetac)(ClO_4)_2]\cdot 4H_2O$ measured at room temperature (red) and one calculated from the single-crystal model (100 K; black).

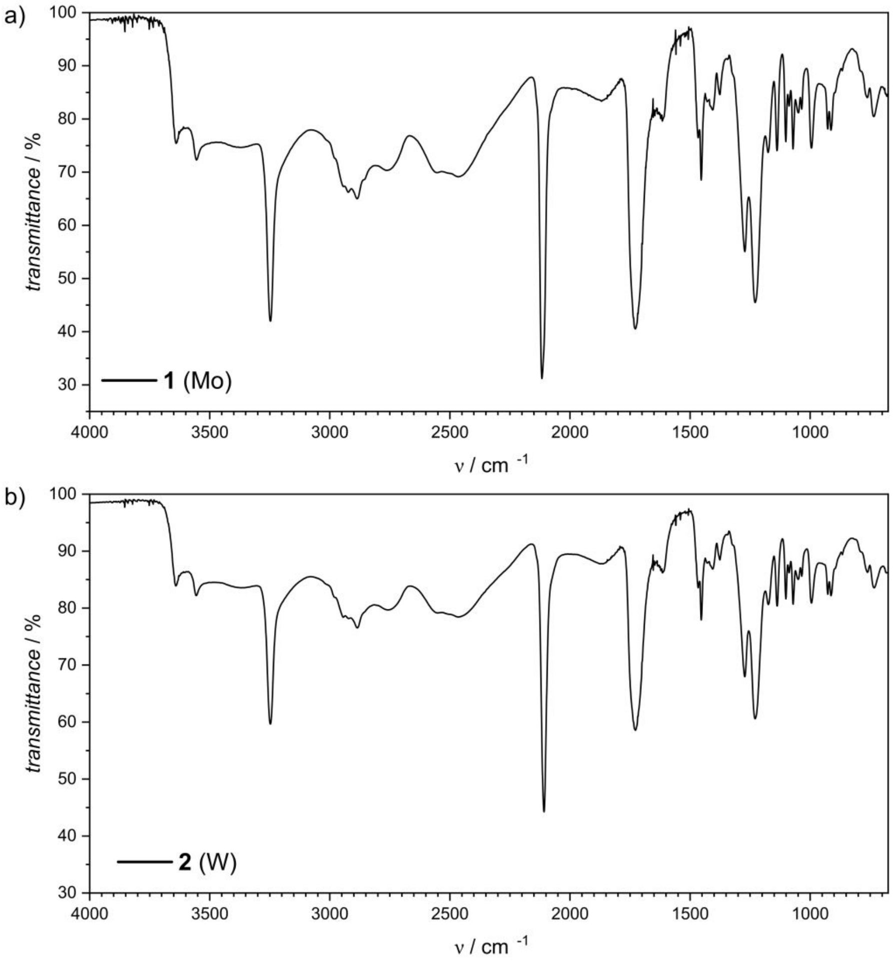

Figure S4. FTIR spectra of **1** (a) and **2** (b).

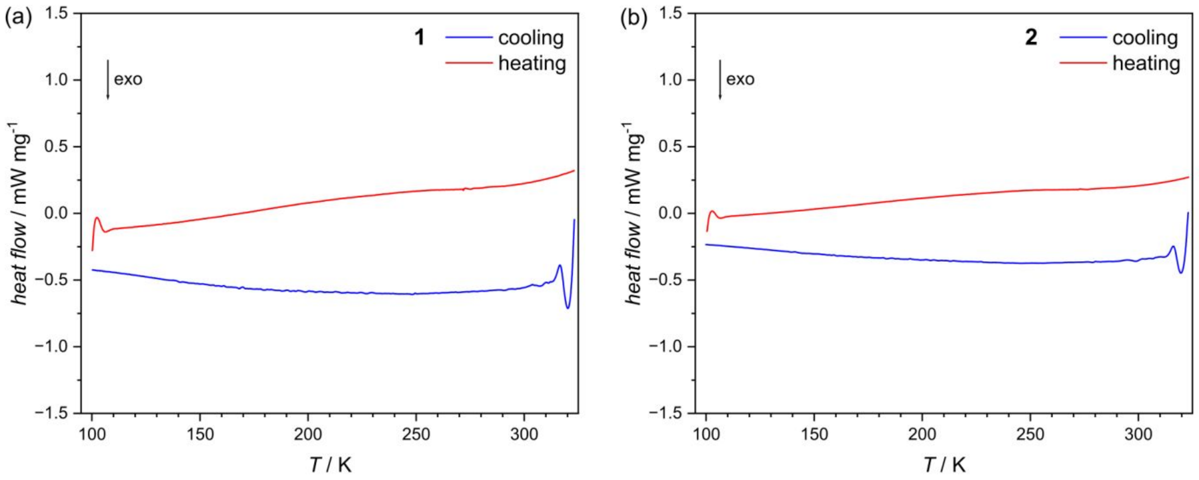

Figure S5. Differential scanning calorimetry profiles of **1** (a) and **2** (b) recorded at 10 K min^{-1} cooling/heating rate.

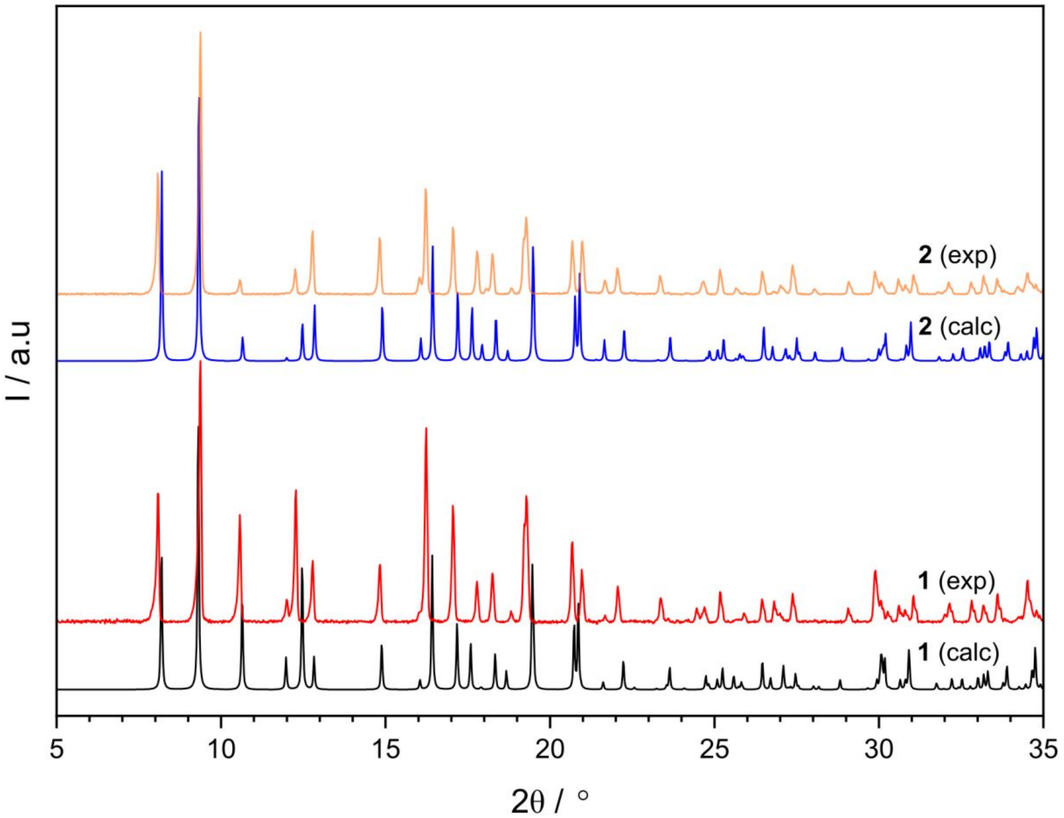

Figure S6. A comparison of powder XRD patterns measured at room temperature (exp) and calculated from the single-crystal model (100 K; calc) for **1** and **2**.

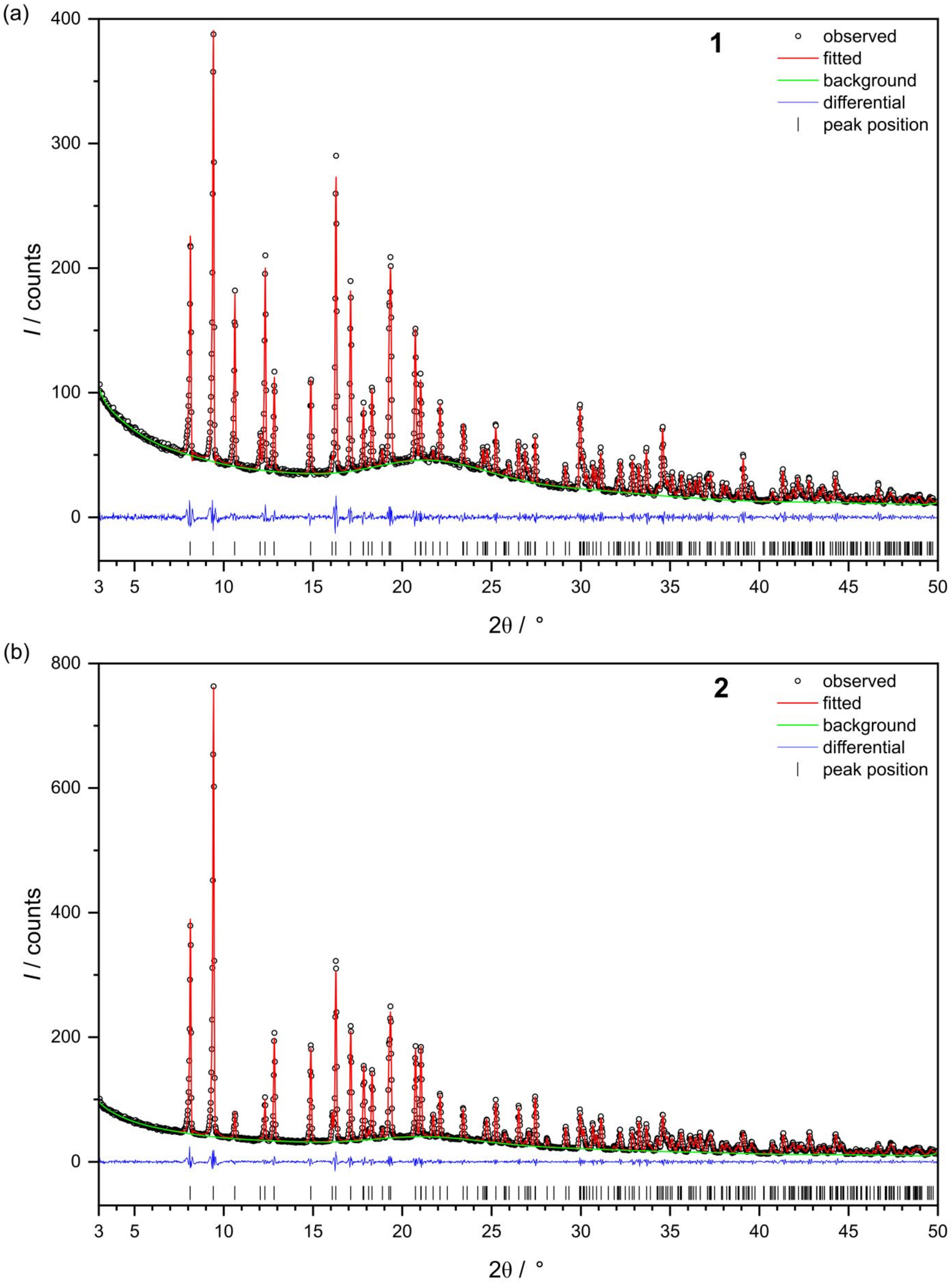

Figure S7. Le Bail profile fitting for room-temperature powder diffraction patterns collected for **1** (a) and **2** (b).

Table S1. La Bail refinement details for room-temperature powder diffraction data of **1** and **2**.

	**1**	**2**
**Formula, molecular weight /g mol⁻¹**	$C_{36}H_{56}N_{16}O_{20}Cu_2Mo$, 1255.96	$C_{36}H_{56}N_{16}O_{20}Cu_2W$, 1343.86
**Radiation source**	Cu Kα, λ = 1.54056 Å	
**system, space group**	orthorhombic, *Fdd*2	
***a* / Å**	10.4189(4)	10.4095(4)
***b* / Å**	28.7357(12)	28.7598(12)
***c* / Å**	33.3267(14)	33.3116(14)
***V* / Å³**	9978.8(7)	9972.7(7)
**2θ range, step / °**	3.0–50.0, 0.02	3.0–50.0, 0.02
**No. of data points**	2316	2316
**No. of Bragg reflections**	227	227
**Peak profile function**	Pseudo-Voigt	
**R_p / %**	3.037	3.238
**R_{wp} / %**	4.148	4.331
**χ^2**	0.069	0.077

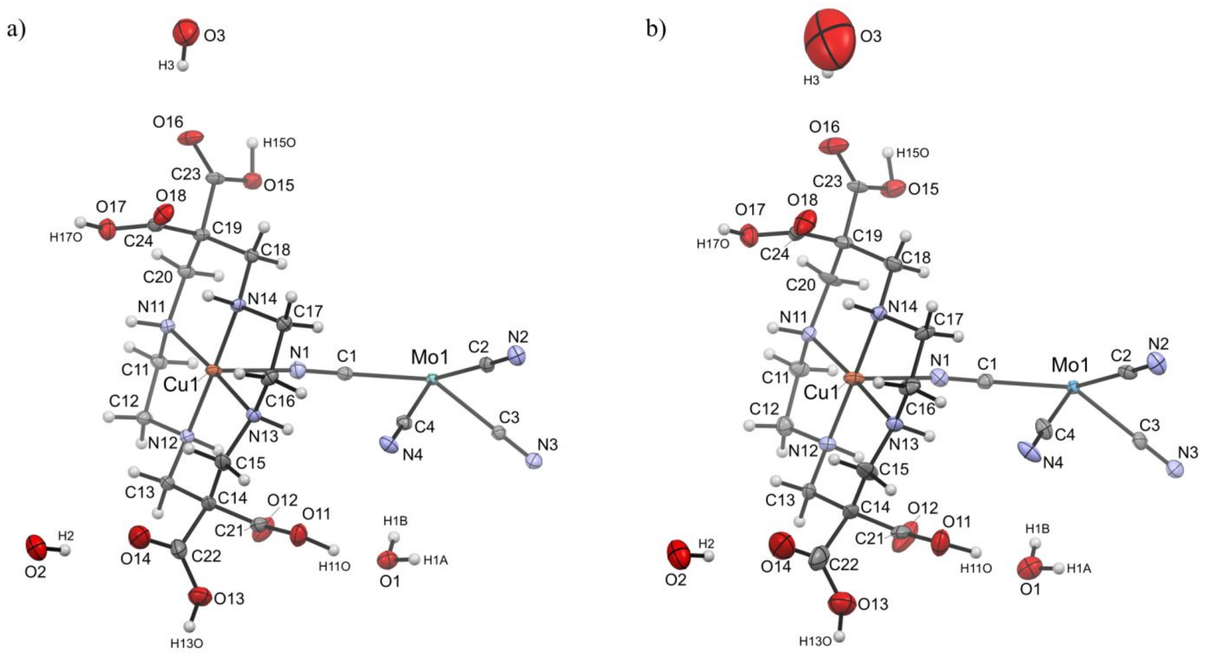

Figure S8. Asymmetric units for **1** and **2**. The anisotropic displacement ellipsoids are drawn with 50% probability.

Table S2. Selected crystal data and details of structure refinement for **1** and **2**.

	**1**	**2**
		crystal data
**formula**	$C_{36}H_{56}Cu_2MoN_{16}O_{20}$	$C_{36}H_{56}Cu_2N_{16}O_{20}W$
M_r **/ g·mol**$^{-1}$	1255.98	1343.89
**radiation type**	Mo *Kα* radiation	
**crystal system**	orthorhombic	orthorhombic
**space group**	*Fdd*2	*Fdd*2
T **/ K**	100(2)	100(2)
a **/ Å**	10.5745(6)	10.5489(4)
b **/ Å**	28.3749(14)	28.3624(11)
c **/ Å**	33.2237(19)	33.1801(14)
V **/ Å**^{3}	9968.8(9)	9927.2(7)
Z	8	8
**calculated density / g·cm**^{3}	1.674	1.798
**μ / mm**$^{-1}$	1.19	3.25
F**(000)**	5152	5408
**crystal size / mm × mm × mm**	0.23 × 0.02 × 0.02	0.19 × 0.04 × 0.02
		data collection
ϑ **range**	2.2°–31.0°	3.0°–27.5°
**limiting indices**	$h = -15 \rightarrow 14$<br>$k = -38 \rightarrow 41$<br>$l = -46 \rightarrow 47$	$h = -14 \rightarrow 14$<br>$k = -40 \rightarrow 39$<br>$l = -46 \rightarrow 46$
**measured reflections**	50959	71189
**independent reflections**	7795	7624
**observed reflections** $I > 2\sigma(I)$	6943	7194
R_{int}	0.059	0.039
**completeness / %**	99.8	99.8
		refinement
**refinement method**	full-matrix least-squares on F^2	
**data / parameters / restraints**	7795 / 348 / 9	7624 / 348 / 11
**GoF on** F^2	1.03	1.11
**final** ***R*** **indices**	$R[F^2 > 2\sigma(F^2)] = 0.040$<br>$wR(F^2) = 0.105$<br>$w = 1/[\sigma^2(F_o^2) + (0.0636P)^2 + 18.1644P]$	$R[F^2 > 2\sigma(F^2)] = 0.028$<br>$wR(F^2) = 0.066$<br>$w = 1/[\sigma^2(F_o^2) + (0.026P)^2 + 83.1905P]$
	$P = (F_o^2 + 2F_c^2)/3$	
**largest diff. peak / hole / e·Å**$^{-3}$	1.06 / –0.54	1.55 / −1.81
**Absolute structure parameter** *(BASF factor for inversion twin refinement)*	0.516(17)	0.520(10)
**CCDC deposition number**	2379259	2379258

Table S3. Selected bond lengths (Å) and angles (°) in the structure of **1** and **2** at 100 K.

	**1** (M = Mo)	**2** (M = W)
M1—C1	2.159(4)	2.176(7)
M1—C2	2.174(4)	2.174(8)
M1—C3	2.151(4)	2.150(8)
M1—C4	2.147(4)	2.144(7)
Cu1—N1	2.407(4)	2.447(7)
Cu1—N2^{i}	2.741(4)	2.702(7)
Cu1—N11	2.029(4)	2.026(6)
Cu1—N12	2.026(4)	2.026(7)
Cu1—N13	2.017(4)	2.021(6)
Cu1—N14	2.016(4)	2.014(6)
C21—O11 (H)	1.316(6)	1.326(8)
C21—O12	1.204(6)	1.213(10)
C22—O13 (H)	1.287(7)	1.268(10)
C22—O14	1.218(7)	1.168(11)
C23—O15 (H)	1.299(6)	1.300(7)
C23—O16	1.222(5)	1.214(7)
C24—O17 (H)	1.316(6)	1.333(8)
C24—O18	1.211(6)	1.168(8)
N1—C1—M1	177.1(4)	176.7(7)
N2—C2—M1	178.0(4)	178.9(6)
N3—C3—M1	177.1(4)	174.5(7)
N4—C4—M1	178.0(4)	178.6(8)
C1—N1—Cu1	149.5(4)	148.8(6)
C2^{i}—N2^{i}—Cu1	141.0(4)	141.5(6)
C1—M1—C1^{ii}	72.4(2)	72.2(4)
C2—M1—C2^{ii}	72.4(2)	72.3(4)
C1—M1—C2	129.76(14)	130.10(16)
N1—Cu1—N2^{i}	177.63(14)	177.8(2)
N1—Cu1—N11	99.40(15)	98.1(2)
N1—Cu1—N12	91.70(15)	91.4(2)
N1—Cu1—N13	85.98(15)	85.6(2)
N1—Cu1—N14	90.32(15)	90.2(2)
N2^{i}—Cu1—N11	82.95(14)	84.0(2)
N2^{i}—Cu1—N12	88.14(14)	88.3(2)
N2^{i}—Cu1—N13	91.67(14)	92.3(2)
N2^{i}—Cu1—N14	89.81(14)	90.1(2)
N11—Cu1—N12	86.33(15)	86.0(2)
N12—Cu1—N13	93.64(16)	93.3(3)
N13—Cu1—N14	85.95(15)	86.4(3)
N14—Cu1—N11	93.88(15)	94.1(2)

Symmetry codes: (i) $x-1/4$, $-y+5/4$, $z-1/4$; (ii) $-x+3/2$, $-y+3/2$, z.

Table S4. Hydrogen-bond geometry in **1** and **2** at 100 K.

D*—H···*A	***D*—H / Å**	**H···*A* / Å**	***D*···*A* / Å**	***D*—H···*A* / °**
				**1**
N11—H11···N2^{ii}	1.00	2.66	3.204(6)	114
N11—H11···N3^{iii}	1.00	2.51	3.293(6)	135
N11—H11···O17	1.00	2.47	3.066(5)	118
N12—H12···O12	1.00	2.32	2.967(5)	122
N13—H13···N4	1.00	2.60	3.419(6)	139
N13—H13···O11	1.00	2.62	3.181(5)	115
N14—H14···O18	1.00	2.28	2.960(5)	124
N14—H14···O18^{iv}	1.00	2.07	2.954(5)	146
O1—H1*A*···O2^{v}	0.84	1.92	2.750(6)	170
O1—H1*B*···N1^{i}	0.84	2.11	2.923(5)	161
O2—H2···O14	0.85	2.01	2.824(6)	161
O3—H3···O16	0.86	2.16	2.981(5)	160
O11—H11*O*···O1	1.04	1.51	2.544(5)	169
O13—H13*O*···N3^{vi}	1.04	1.60	2.623(6)	164
O15—H15*O*···N4^{vii}	1.01	1.71	2.669(5)	156
O17—H17*O*···N2^{iii}	1.01	2.01	2.941(5)	153
				**2**
N11—H11···N2^{ii}	1.00	2.68	3.204(9)	113
N11—H11···N3^{iii}	1.00	2.50	3.292(9)	136
N11—H11···O17	1.00	2.48	3.097(7)	119
N12—H12···O12	1.00	2.33	2.981(9)	122
N12—H12···O12^{i}	1.00	2.65	3.443(9)	136
N13—H13···N4	1.00	2.58	3.398(10)	139
N13—H13···O11	1.00	2.64	3.158(8)	112
N14—H14···O18	1.00	2.27	2.945(7)	124
N14—H14···O18^{iv}	1.00	2.06	2.935(7)	145
O1—H1*A*···O2^{v}	0.85	1.89	2.740(8)	178
O1—H1*B*···N1^{i}	0.85	2.09	2.898(8)	158
O2—H2···O14	0.85	2.00	2.833(8)	168
O3—H3···O16	0.85	2.18	3.000(14)	163
O11—H11*O*···O1	1.00	1.54	2.541(7)	179
O13—H13*O*···N3^{vi}	1.02	1.70	2.623(9)	148
O15—H15*O*···N4^{vii}	1.01	1.69	2.656(9)	159
O17—H17*O*···N2^{iii}	1.00	2.05	2.983(8)	154

Symmetry codes: (i) −*x*+3/2, −*y*+3/2, *z*; (ii) *x*−1/4, −*y*+5/4, *z*−1/4; (iii) −*x*+5/4, *y*−1/4, *z*−1/4; (iv) −*x*+1, −*y*+1, *z*; (v) *x*+1/4, −*y*+5/4, *z*+1/4; (vi) -*x*+9/4, *y*−1/4, *z*−1/4; (vii) *x*−1, *y*, *z*.

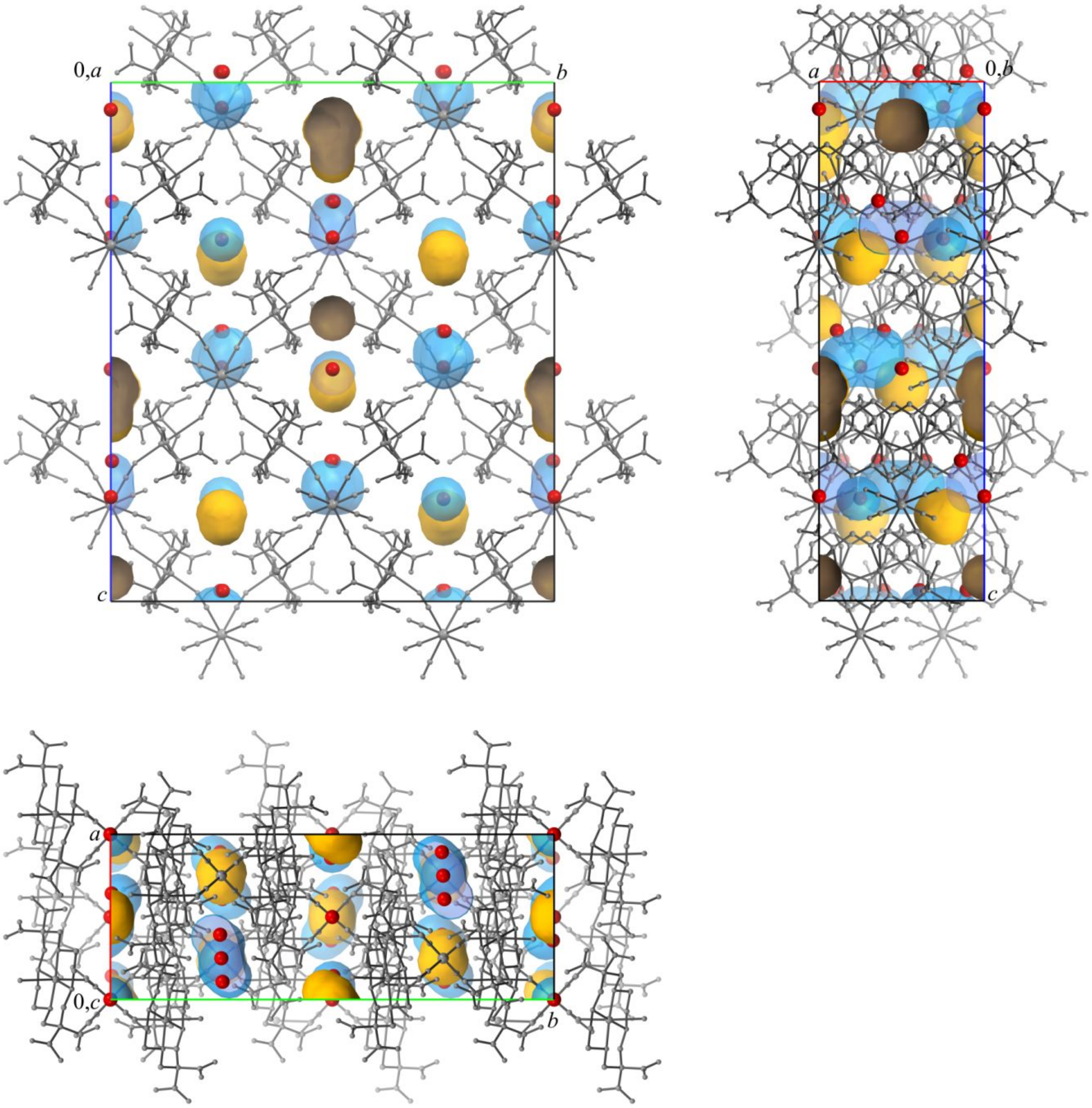

Figure S9. Packing diagrams for **1** presenting solvent accessible volume (calculated with Mercury4.0 for probe radius = 1.4 Å and grid spacing = 0.5 Å).[2] Blue contours represent voids occupied by crystallization water molecules (red spheres). Orange contours represent cavities in which no water molecules were localized.

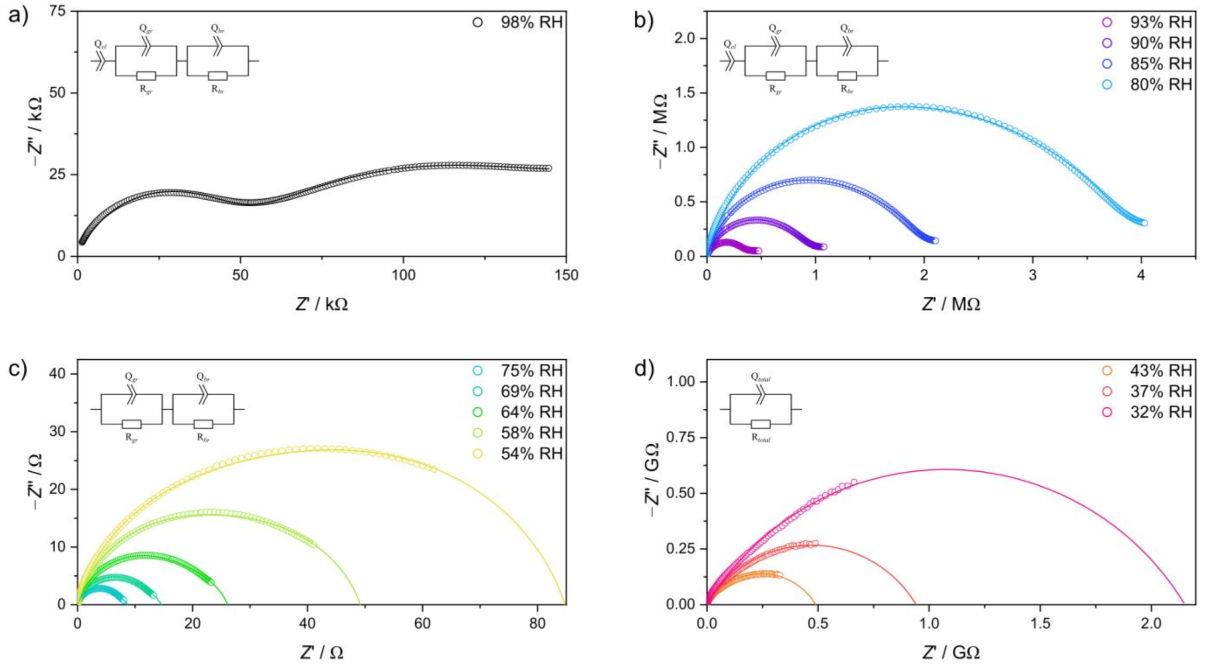

Figure S10. Nyquist plots for **1** collected at various relative humidity at 298 K. The solid lines represent fits of equivalent circuits given in the inserts.

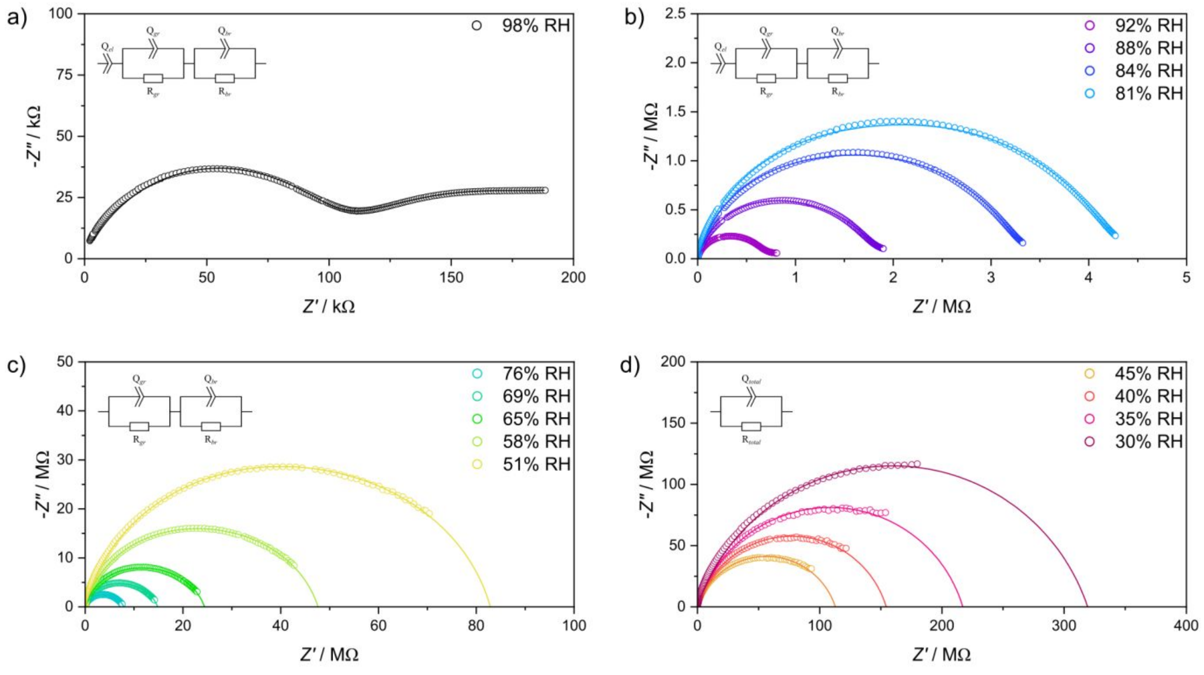

Figure S11. Nyquist plots for **2** collected at various relative humidity at 298 K. The solid lines represent fits of equivalent circuits given in the inserts.

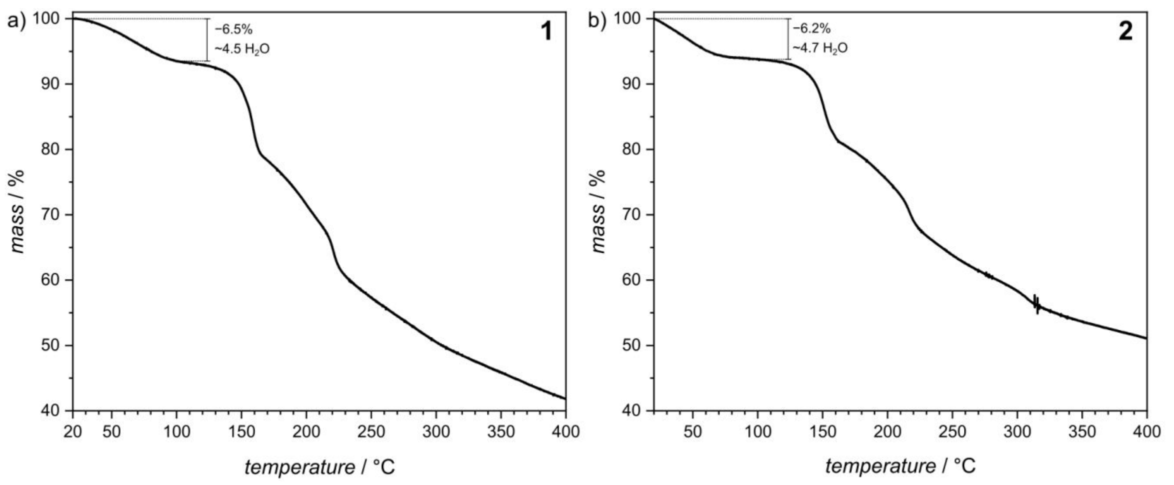

Figure S12. Thermogravimetric profiles for **1** (a) and **2** (b) were recorded at the heating ratio of 1 °C/min in the flow of dry N_2.

Comment to Figure S12. The TGA profiles show that the dehydration of both materials starts slightly above the room temperature in dry N_2. The mass changes recorded up to 100 °C correspond with a release of slightly more water molecules per formula unit than could be expected based on the crystal structure (4 H_2O). Further heating above 130 °C leads to the decomposition of the materials.

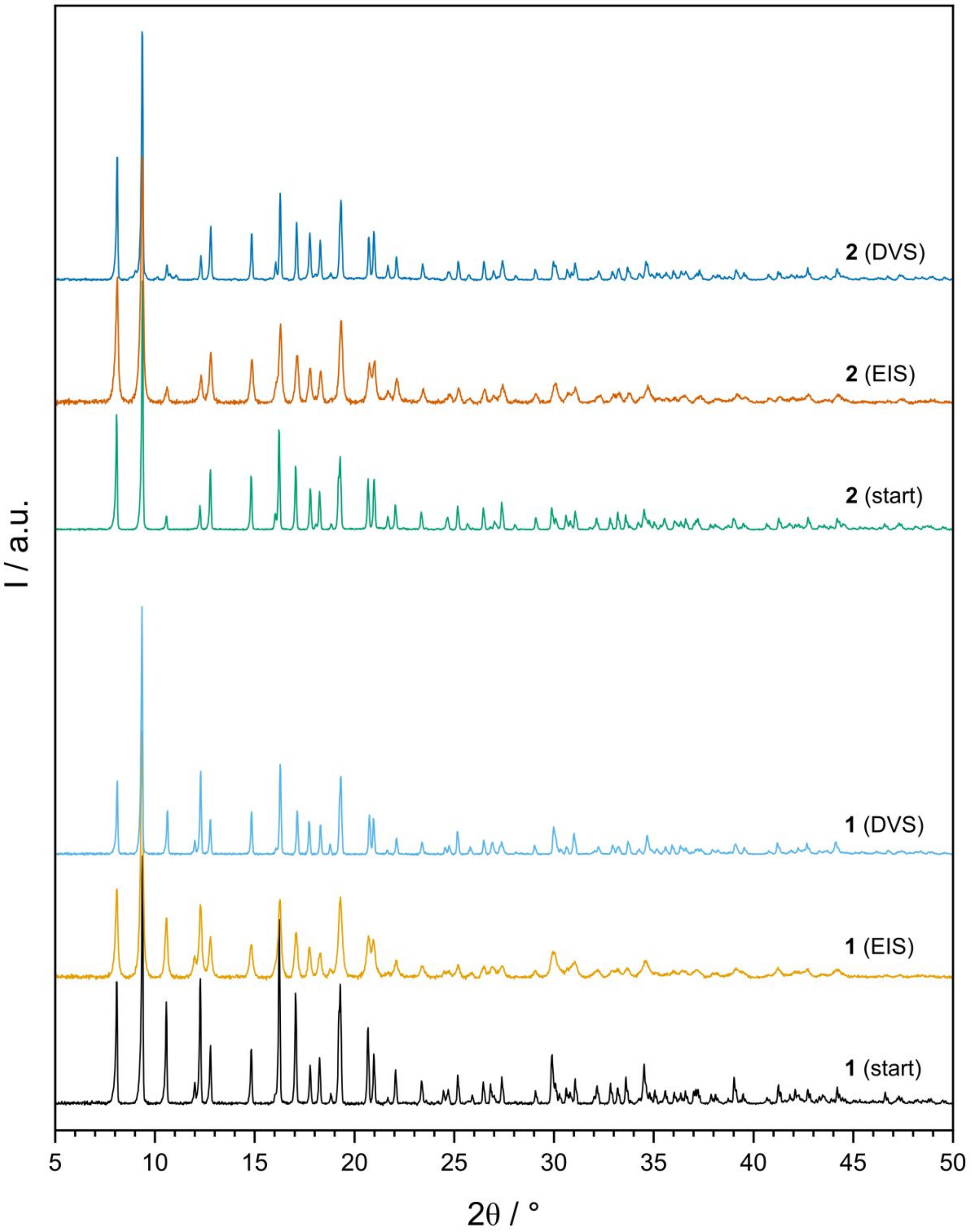

Figure S13. PXRD patterns recorded for **1** and **2** before (start) and after the electrochemical impedance spectroscopy (EIS) and dynamic vapor sorption (DVS) experiments. The broadening of diffraction peaks after EIS measurements comes from the sample preparation process (powdering and pressing into a pellet).

Table S5. Proton conductivity of selected COOH-based coordination systems around room temperature.

compound	conditions	σ / S cm^{-1}	E_a / eV	ref
**1**	298 K, 98% RH	5.4×10^{-7}	0.37	this work
**2**	298 K, 98% RH	8.6×10^{-7}	0.37	this work
$Rb_2(adp)[Zn_2(ox)_3]\cdot 3H_2O$	298 K, 98% RH	4.3×10^{-5}	0.69	4
$K_2(adp)[Zn_2(ox)_3]\cdot 3H_2O$	298 K, 98% RH	1.2×10^{-4}	0.63	5
$(NH_4)_2(adp)[Zn_2(ox)_3]\cdot 3H_2O$	298 K, 98% RH	8×10^{-2}	0.63	6
MIL-53(Fe)	298 K, 95% RH	8×10^{-6}	0.21	7
$Fe(OH)(BDC\text{-}(COOH)_2)$				
UiO-66(Zr)-(COOH)	303 K, 97% RH	1×10^{-3}	0.18	8
$Zr_6O_4(OH)_4(BDC\text{-}(COOH)_2)$				
$[Co(COOH\text{-}terpy)_2](ClO_4)_2\cdot 4H_2O$	298 K, 98% RH	1.32×10^{-4}	0.44	9

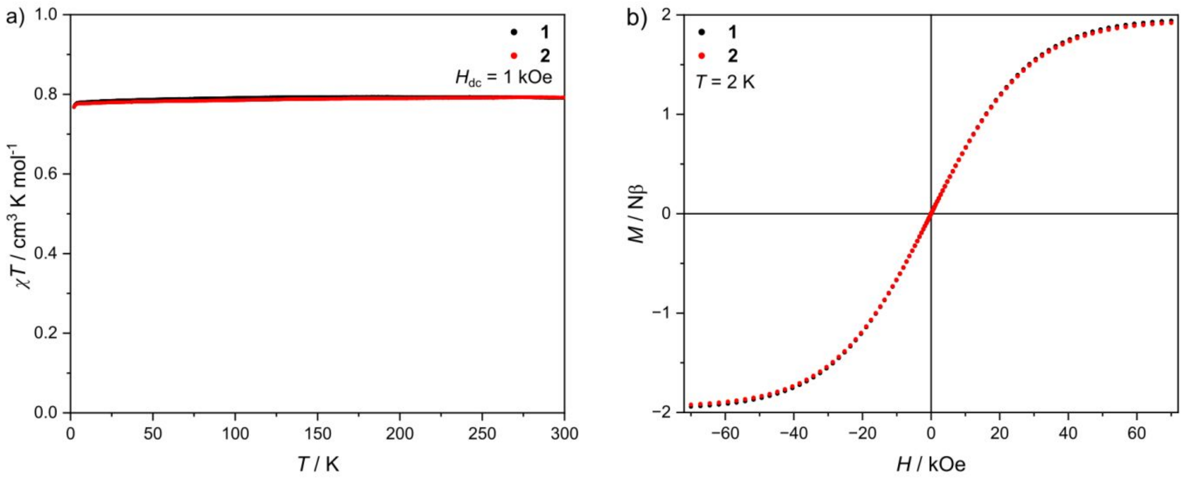

Figure S14. Magnetic properties of bulk samples of **1** (black) and **2** (red): (a) temperature dependence of magnetic susceptibility and temperature (χT) at external dc field of 1 kOe and (b) field dependence of magnetization (M) at 2 K.

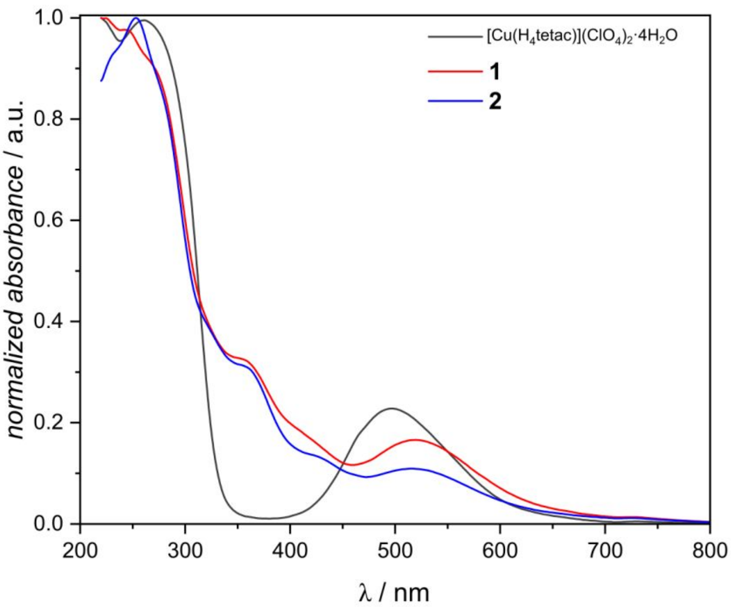

Figure S15. Solid-state UV-vis spectra of **1** and **2** compared with the spectrum of $[Cu(H_4tetac)](ClO_4)_2 \cdot 4H_2O$. Compared to the precursor, the Cu-centered ligand-field bands in **1** and **2** are red-shifted and the absorbance around 450 nm mostly comes from ligand-field transitions in $[M^{IV}(CN)_8]^{4-}$.

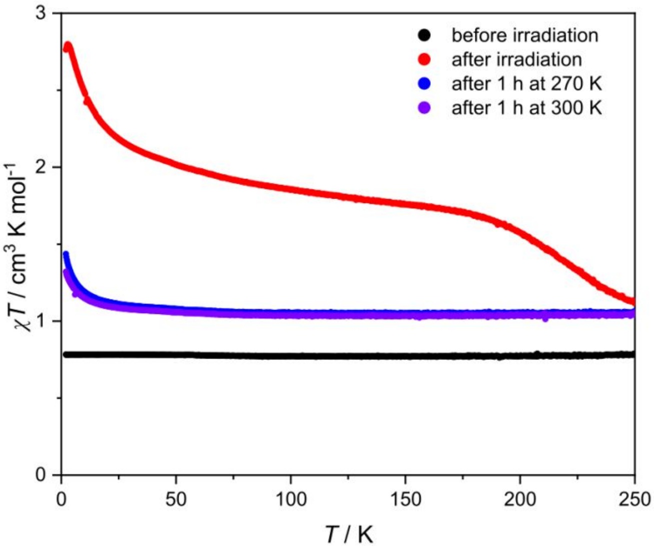

Figure S16. Temperature dependence of χT at 1 kOe recorded for **1** before light irradiation (black points), after 450 nm light irradiation (red points), and after thermalization at 270 K (blue points) or 300 K (purple points).

REFERENCES

(1) Xin, L.; Curtis, N. F.; Weatherburn, D. C. Compounds of copper(II) and nickel(II) with 6,6,13,13-tetracarboxy-(and *E*-6,13-dicarboxy-) substituted 1,4,8,11-tetrazacyclotetradecanes, and carbomethoxy- and carbethoxy- derivatives. Structures of two isomeric *E*-6,13-dicarboxy- (and an *E*-6,13-dicarbomethoxy-)1,4,8,11-tetrazacyclotetradecane copper(II) perchlorates. *Transition Met. Chem.* **1992**, *17* (2), 147-154. DOI: 10.1007/BF02910808.

(2) Macrae, C. F.; Sovago, I.; Cottrell, S. J.; Galek, P. T. A.; McCabe, P.; Pidcock, E.; Platings, M.; Shields, G. P.; Stevens, J. S.; Towler, M.; et al. Mercury 4.0: from visualization to analysis, design and prediction. *J. Appl. Cryst.* **2020**, *53* (1), 226-235. DOI: 10.1107/S1600576719014092.

(3) Thommes, M.; Kaneko, K.; Neimark Alexander, V.; Olivier James, P.; Rodriguez-Reinoso, F.; Rouquerol, J.; Sing Kenneth, S. W. Physisorption of gases, with special reference to the evaluation of surface area and pore size distribution (IUPAC Technical Report). In *Pure and Applied Chemistry*, 2015; Vol. 87, p 1051.

(4) Sadakiyo, M.; Yamada, T.; Kitagawa, H. A study on proton conduction in a layered metal–organic framework, $Rb_2(adp)[Zn_2(ox)_3]\cdot 3H_2O$ (adp=adipic acid, ox^{2-}=oxalate). *Inorg. Chem. Commun.* **2016**, *72*, 138-140. DOI: 10.1016/j.inoche.2016.08.016.

(5) Sadakiyo, M.; Yamada, T.; Kitagawa, H. Proton Conductivity Control by Ion Substitution in a Highly Proton-Conductive Metal–Organic Framework. *J. Am. Chem. Soc.* **2014**, *136* (38), 13166-13169. DOI: 10.1021/ja507634v.

(6) Sadakiyo, M.; Yamada, T.; Kitagawa, H. Rational Designs for Highly Proton-Conductive Metal−Organic Frameworks. *J. Am. Chem. Soc.* **2009**, *131* (29), 9906-9907. DOI: 10.1021/ja9040016.

(7) Shigematsu, A.; Yamada, T.; Kitagawa, H. Wide Control of Proton Conductivity in Porous Coordination Polymers. *J. Am. Chem. Soc.* **2011**, *133* (7), 2034-2036. DOI: 10.1021/ja109810w.

(8) Yang, F.; Huang, H.; Wang, X.; Li, F.; Gong, Y.; Zhong, C.; Li, J.-R. Proton Conductivities in Functionalized UiO-66: Tuned Properties, Thermogravimetry Mass, and Molecular Simulation Analyses. *Cryst. Growth Des.* **2015**, *15* (12), 5827-5833. DOI: 10.1021/acs.cgd.5b01190.

(9) Kobayashi, F.; Hiramatsu, T.; Sueyasu, K.; Tadokoro, M. Proton Conductive Mononuclear Hydrogen-Bonded Cobalt(II) Spin Crossover Complex. *Cryst. Growth Des.* **2023**, *23* (3), 1633-1640. DOI: 10.1021/acs.cgd.2c01243.
